# Neurophysiological and Genetic Findings in Patients With Juvenile Myoclonic Epilepsy

**DOI:** 10.3389/fnint.2020.00045

**Published:** 2020-08-20

**Authors:** Stefani Stefani, Ioanna Kousiappa, Nicoletta Nicolaou, Eleftherios S. Papathanasiou, Anastasis Oulas, Pavlos Fanis, Vassos Neocleous, Leonidas A. Phylactou, George M. Spyrou, Savvas S. Papacostas

**Affiliations:** ^1^Cyprus School of Molecular Medicine, Nicosia, Cyprus; ^2^Neurology Clinic B, The Cyprus Institute of Neurology and Genetics, Nicosia, Cyprus; ^3^Medical School, University of Nicosia, Nicosia, Cyprus; ^4^Centre for Neuroscience and Integrative Brain Research (CENIBRE), University of Nicosia, Nicosia, Cyprus; ^5^Bioinformatics Group, The Cyprus Institute of Neurology and Genetics, Nicosia, Cyprus; ^6^Department of Molecular Genetics, Function & Therapy, The Cyprus Institute of Neurology and Genetics, Nicosia, Cyprus

**Keywords:** juvenile myoclonic epilepsy, transcranial magnetic stimulation, neurophysiology, polyphasia, genetics, whole exome sequencing, polymorphism

## Abstract

**Objective:**

Transcranial magnetic stimulation (TMS), a non-invasive procedure, stimulates the cortex evaluating the central motor pathways. The response is called motor evoked potential (MEP). Polyphasia results when the response crosses the baseline more than twice (zero crossing). Recent research shows MEP polyphasia in patients with generalized genetic epilepsy (GGE) and their first-degree relatives compared with controls. Juvenile Myoclonic Epilepsy (JME), a GGE type, is not well studied regarding polyphasia. In our study, we assessed polyphasia appearance probability with TMS in JME patients, their healthy first-degree relatives and controls. Two genetic approaches were applied to uncover genetic association with polyphasia.

**Methods:**

20 JME patients, 23 first-degree relatives and 30 controls underwent TMS, obtaining 10–15 MEPs per participant. We evaluated MEP mean number of phases, proportion of MEP trials displaying polyphasia for each subject and variability between groups. Participants underwent whole exome sequencing (WES) via trio-based analysis and two-case scenario. Extensive bioinformatics analysis was applied.

**Results:**

We identified increased polyphasia in patients (85%) and relatives (70%) compared to controls (47%) and significantly higher mean number of zero crossings (i.e., occurrence of phases) (patients 1.49, relatives 1.46, controls 1.22; *p* < 0.05). Trio-based analysis revealed a candidate polymorphism, p.Glu270del,in *SYT14 (Synaptotagmin 14)*, in JME patients and their relatives presenting polyphasia. Sanger sequencing analysis in remaining participants showed no significant association. In two-case scenario, a machine learning approach was applied in variants identified from odds ratio analysis and risk prediction scores were obtained for polyphasia. The results revealed 61 variants of which none was associated with polyphasia. Risk prediction scores indeed showed lower probability for non-polyphasic subjects on having polyphasia and higher probability for polyphasic subjects on having polyphasia.

**Conclusion:**

Polyphasia was present in JME patients and relatives in contrast to controls. Although no known clinical symptoms are linked to polyphasia this neurophysiological phenomenon is likely due to common cerebral electrophysiological abnormality. We did not discover direct association between genetic variants obtained and polyphasia. It is likely these genetic traits alone cannot provoke polyphasia, however, this predisposition combined with disturbed brain-electrical activity and tendency to generate seizures may increase the risk of developing polyphasia, mainly in patients and relatives.

## Introduction

Epilepsy is a common neurological disorder that can affect people of all ages and is underlined by a functional disturbance of the brain (Fisher et al., 2014). It is a chronic and heterogeneous condition characterized by disturbed brain electrical activity and predisposition to generating repeating seizures (> 24 h apart) due to increased synchronicity of neuronal networks (Engelborghs et al., 2000; Guerreiro, 2016). Juvenile myoclonic epilepsy (JME), a type of genetic generalized epilepsy (GGE) affecting adolescents and adults, represents 2.8–11.9% of the epilepsies and 26.7% of GGE (Yacubian, 2017). It is characterized by generalized tonic-clonic seizures, myoclonic jerks and absence seizures where the patient loses consciousness (Guaranha et al., 2011; Carvalho et al., 2016; Brodie et al., 2018). In the majority of cases seizures start during mid to late childhood, between 12 and 18 years of age and can be triggered by sleep-deprivation, alcohol, and medication non-compliance (Guaranha et al., 2011; Carvalho et al., 2016; Baykan and Wolf, 2017).

Epilepsy has strong genetic underpinnings and some syndromes have been characterized genetically with ∼70–80% of the cases caused by specific genetic mutations (Lemke et al., 2012; Myers and Mefford, 2015). JME is considered to have a complicated and heterogeneous genetic background (Wolf et al., 2015). Although attempts were made to discover related genes, they still remain elusive for the majority of patients (Baykan and Wolf, 2017). Some genes were recognized as causative for JME: *CACNB4, EFHC1, GABRA1, CLCN2*, *and GABA-A*, with the majority of them coding for ion channels (Delgado-Escueta, 2007; Delgado-Escueta et al., 2013; Arain et al., 2015; Bailey et al., 2017; Wang et al., 2017). It has also been suggested that some single nucleotide polymorphism alleles are involved in *ME2, BRD2*, and *Cx*-*36*, and some copy number variations but this has yet to be confirmed (Delgado-Escueta et al., 2013). Whole Exome Sequencing (WES) can be used to investigate genome protein-coding regions and reveal genetic influences on complex genetic diseases, like JME and other epilepsy syndromes. Although the exome represents < 2% of the genome, it contains ∼85% of known disease-associated variants.

The electroencephalogram (EEG) of JME patients is characterized by irregular polyspike and wave complexes triggered by photic stimulation (Kasteleijn-Nolst Trenite et al., 2013; Serafini et al., 2013; Seneviratne et al., 2017) but individual EEG variability may hinder diagnosis. Symptoms usually appear upon awakening in the morning or after a nap and are commonly accompanied by myoclonic jerks (Baykan and Wolf, 2017). Myoclonic jerks usually constitute a warning of an impending generalized tonic-clonic seizure (Baykan and Wolf, 2017). While generalized tonic-clonic seizures occur in almost all patients, absence seizures are only present in ∼1 out of 3 JME patients (Baykan and Wolf, 2017).

Transcranial Magnetic Stimulation (TMS) is a painless, non-invasive neurophysiological technique stimulating the cortex without any current passing through the skin or the meninges of skull where most of the pain nerves are found (Barker et al., 1985; Hameed et al., 2017; Starnes et al., 2019). There is generation of intracranial currents that can alter cortical excitability, evoking action potentials called Motor Evoked Potentials (MEP) and also providing information about synaptic interactions between neurons and local neuronal (Rossini and Rossi, 2007; Rossi et al., 2009b; Starnes et al., 2019). Results from TMS can illustrate connections between interneurons and motor neurons and discern biomarkers that can provide clues about cortical mechanisms involved in excitation and inhibition of the brain (Rothwell, 1997; Tsuboyama et al., 2019). Over the past few decades, TMS has witnessed significant development and has contributed considerably to the field of clinical neurophysiology (Rossi et al., 2009a).

The definitions of polyphasia vary in current literature. Some studies define polyphasia as the presence of more than two phases while others suggest that polyphasia is present when more than four phases are visible (Rodriguez-Carreno et al., 2012; Chowdhury et al., 2015). Given that in most instances a normal MEP has 2 phases, we defined polyphasia as the presence of more than two phases in the post-TMS stimulus MEP (Chowdhury et al., 2015).

Previous studies reported polyphasic Motor Evoked Potential (MEP) in a few diseases. In patients with Amyotrophic Lateral Sclerosis (ALS) and patients with Myoclonus Dystonia (MD) a more complicated waveform with more phases appeared compared to controls (Kohara et al., 1999; Van Der Salm et al., 2009). Moreover, studies showed that non syndromic relatives of patients with idiopathic generalized epilepsy (IGE) had abnormalities on their EEG and MEPs generated following TMS stimulation suggesting a common neurophysiological marker that may characterize a genetically inherited predisposition to epilepsy (Akgun et al., 2009; Badawy et al., 2013). The use of TMS examination in patients with different types of GGE has demonstrated increased polyphasia compared to their healthy first degree relatives and controls (Chowdhury et al., 2015). Chowdhury and colleagues described polyphasia as an electrophysiological characteristic associated with cortical pathophysiology in patients’ relatives who did not have epilepsy (Chowdhury et al., 2015).

In this study the first aim is to investigate whether polyphasia can be observed in MEPs, following cortical TMS stimulation in patients with JME and their first-degree relatives compared to healthy controls. No genetic analysis has been conducted to date investigating the possible correlation of polymorphisms associated with polyphasia in JME. Therefore, the second objective was to implement WES in the study subjects in an effort to identify risk genes and rare variants by using two approaches, a trio-based analysis and a two-case scenario.

## Materials and Methods

### Subjects

The study was approved by the Cyprus National Bioethics Committee (EEBK/EΠ/2016/33) and written informed consent was obtained from each subject before enrollment. Participants were separated in three groups. The first group comprised 20 patients (10 female, 10 male; mean age ± SD: 30.85 ± 9.16; range: 19–50) with a specific JME phenotype recruited from the Epilepsy Clinic of the Cyprus Institute of Neurology and Genetics (CING). The second group consisted of the patients’ 23 first-degree relatives without any neurological diagnoses and with no consanguinity (14 female, 9 male; mean age ± SD: 47.52 ± 15.32; range: 18–65). Relatives were parents, children or siblings of the patients. Thirty healthy individuals (16 female, 14 male; mean age ± SD: 33.33 ± 7.09; range: 19–50) with no family history of seizures, symptoms or signs of any neurological disease comprised the third group (controls). There was no statistical difference between the groups with regards to sex or age (*p* < 0.05).

The clinical characteristics of the patients are summarized in Table 1 and details on the age of relatives and controls are shown in Supplementary Table S1. None of the participants had any blood borne disease, any medical implant or other serious medical condition. With the exception of the group of patients who were taking antiepileptic drugs at the time of the examination, controls and relatives did not take any medication that could affect their central nervous system excitability.

**TABLE 1:** Summary of clinical characteristics of JME patients.

Patient ID	Age range	Age of onset	Seizure frequency			Medication	EEG	MRI/CT scan
			Generalized tonic-clonic	Myoclonic	Absence			
01	20–25	6	Frequent	Rare	Common	LML/KEP/LTG/VPA	GPW/GSW	Normal/Normal
02	40–45	15	Very frequent	Rare	/	VPA	GPW/Ph	Normal/Low Density Area in left temporal lobe
03	30–35	16	Frequent	Frequent	Frequent	LTG	GSW/Ph	Mild constitutional widening of lateral ventricles, cisterna magna/N/A
04	20–25	13.5	Infrequent	Frequent	/	LEV/KEP	GSW/GPW	Normal/N/A
05	20–25	15	Frequent	Rare	Rare	LEV/VPA	GSW/GPW	Normal/N/A
06	35–40	19	Frequent	Rare	/	LTG/OXC	GSW/GPW	Normal/N/A
07	35–40	13	/	Frequent	/	VPA/TPM	GSW/GPW/Ph	Left-slight sided sylvian atrophy/N/A
08	25–30	7	Infrequent	Common	Rare	DEP	GSW/GPW	Normal/N/A
09	30–35	17	Frequent	Common	Common	VPA/LTG	GPW	Normal/N/A
10	35–40	17	/	Rare	Common	/	GSW/GPW	Normal/Normal
11	25–30	15	Infrequent	Common	/	DEP	GSW/GPW	Normal/N/A
12	25–30	18	/	/	/	/	GPW	Normal/N/A
13	45–50	23	/	Rare	Rare	TPM/CBZ	GPW	Normal/N/A
14	20–25	18	Infrequent	Rare	/	/	GSW/GPW	N/A/Normal
15	20–25	15	Infrequent	Frequent	/	DEP	GSW/GPW	Normal/N/A
16	30–35	11	Frequent	Frequent	Rare	VPA	GSW/Ph	Normal/Normal
17	45–50	12	/	/	/	VPA/TPM/LTG	Ph	N/A/(Normal)
18	20–25	16	Frequent	Rare	/	/	GPW	Normal/N/A
19	20–25	17	Infrequent	Rare	/	/	GPW	Normal/N/A
20	20–25	10	Infrequent	Rare	Rare	VPA/	GSW/GPW	Normal/N/A

**CBZ, Carbamazepine; CBZ, Clobazam; DEP, Depakine; GPW, Generalized polyspike and wave; GSW, Generalized spike and wave; JME, Juvenile Myoclonic Epilepsy; KEP, Keppra; LEV, Levetiracetam; LML, Lamictal; LTG, Lamotrigine; Nb, Number; N/A, Non available; OXC, Oxcarbazepine; Ph, Photosensitivity; TPM, Topiramate; VPA, Valproic Acid. Frequency: Infrequent: < 3, Very Frequent: > 15, Frequent: 4–15, Common: weekly-monthly, Rare: < per month.**

**FIGURE 1:**
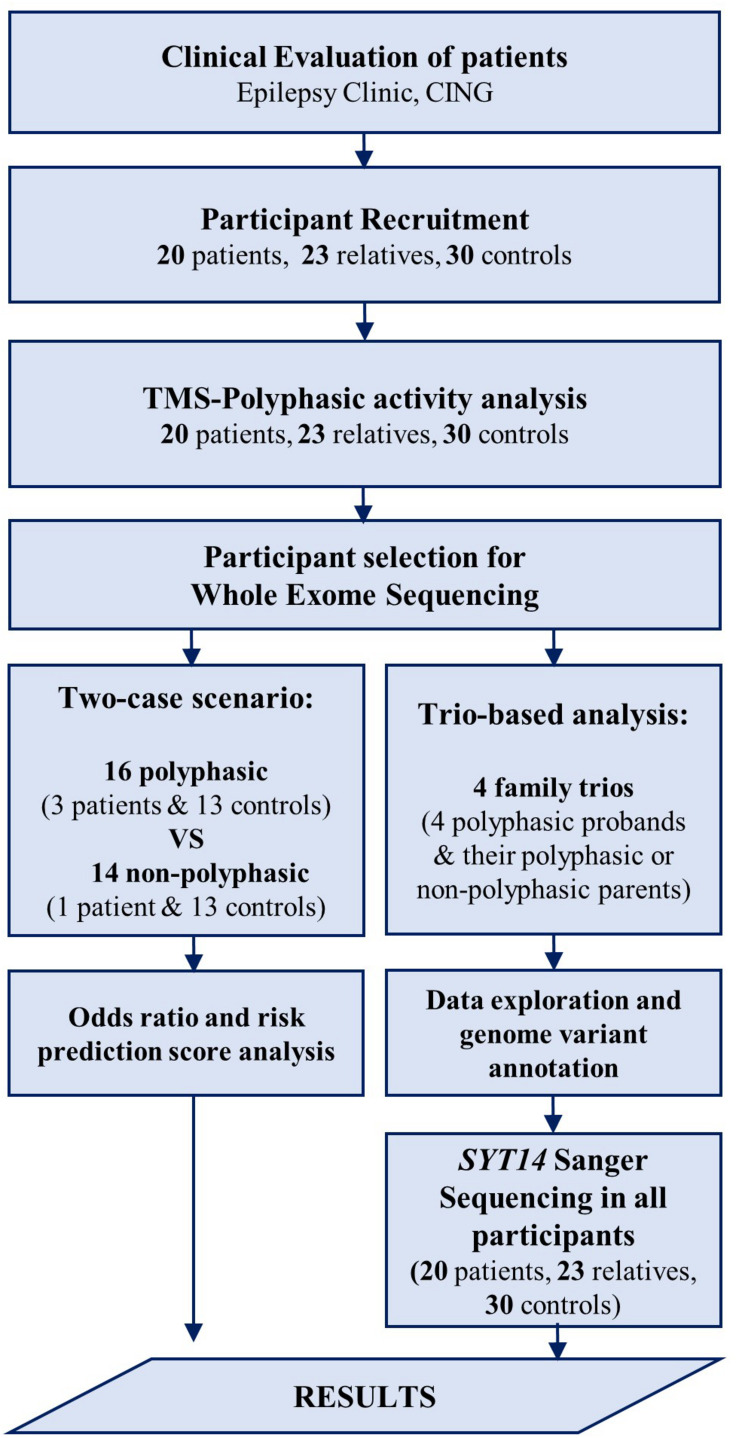
Flow chart of the procedures performed in the study. After the clinical evaluation of patients at the Epilepsy Clinic of CING and the recruitment of the study participants, TMS examination was performed followed by polyphasic activity analysis in all participants. Some of the participants were then selected for WES analysis using two approaches. First, a trio-based analysis performed in four family trios followed by data exploration and genome variant annotation revealing a candidate variant in *SYT14* gene. Sanger sequencing was performed in *SYT14* gene in all study participants. Second, a two-case scenario analysis performed in 16 polyphasic subjects and 14 non-polyphasic subjects followed by odds ratio and risk prediction score analysis. Finally, the results obtained from all investigations done in this study (i.e., neurophysiological examination (TMS) and genetic analysis (WES) were assessed.

Participants underwent the following examinations: TMS and WES. EEG was also performed in the group of patients and relatives, but not in controls (data not shown). EEG was conducted in order to exclude other syndromes of epilepsy for the patients, and make sure that none of the relatives have any type of epilepsy. For the groups of patients and relatives, EEG recording and TMS examination were conducted on the same day, either in the morning or early in the afternoon, and individuals were not sleep deprived. The flow chart (Figure 1) clarifies the different procedures performed in this study and Table 2 includes the number of participants that are involved.

**TABLE 2:** Number of participants included in the different parts of the study.

Participants	Neuro physiological examination	Whole exome sequencing		Sanger sequencing
		
	TMS	Trio-based analysis	Two-case scenario analysis	
Patients	20	4	4	20
First-degree relatives	23	8	0	23
Healthy individuals	30	0	26	30

### Neurophysiological Examination

MEP recordings following TMS were obtained in a single session. Once the participants were relaxed and seated in a chair, surface electromyography recordings were obtained from both sides of the body successively, one after the other (right and then left) with reusable surface disk electrodes, placed on the muscle being examined; abductor digiti minimi (ADM) for the hand and tibialis anterior (TA) for the leg. The hand muscles were examined from each side, followed by the leg muscles for each side. The active electrode was placed on the muscle belly and the reference electrode on the tendon of the same muscle. The ground electrode was placed on the hand wrist (carpal bone joint) and tibia lower bone during hand and leg stimulation, respectively. Initially, peripheral electrical stimulation was performed stimulating the ulnar nerve at the wrist and the peroneal nerve in the popliteal fossa. TMS single-pulses were given by a circular coil (C-100) with an inner diameter of 20 mm and outer diameter of 110 mm and a butterfly coil (C-B60) with an inner diameter of 35 mm and an outer diameter of 75 mm connected to the MagVenture, MagPro R20 machine (MagVenture User Guide, United Kingdom edition, MagVenture A/S, Denmark). Traces were recorded with the help of the KeyPoint.Net Software Electromyography using a bandwidth of 20 Hz–10 kHz, with 5 ms/D for the sweep and 5 mV/D for the sensitivity with a single-pulse stimulation frequency.

The central motor cortex and spinal cord were stimulated with TMS. The coil was placed tangentially to the head for the cortical stimulation, with the center of the coil placed over the vertex in order to produce a proper and favorable stimulation and the current to flow all over the motor cortex and over the C8 and L4 spinous process for cervical and lumbar stimulation, respectively. The stimulation was performed separately, first over the motor cortex and then over the spinal cord (Rossini et al., 2015).

All individuals in the groups were examined from the active ADM and TA muscles with a contraction force of 20–30% of maximum voluntary contraction. A total of 10–15 pulses were recorded for each subject. Stimulation started at 40% of the maximum output and increased by 5% increments until a maximum MEP was obtained. Auditory feedback was provided to the participants in order to maintain the right level of contraction. The testing session lasted 40–50 min and all data were saved for offline analysis using the computerized software Keypoint.Net Software, Denmark (Dantec Enterprise).

The MEPs were inspected and the collected data were evaluated regarding the number of phases in each individual MEP. Given that in most instances a normal EMG has 2 phases, we defined polyphasia as the presence of more than two phases in the post-TMS stimulus MEP (Chowdhury et al., 2015).

The analysis of MEPs for polyphasia was conducted in GNU Octave (version 5.1.0). Initially, MEP responses were visually inspected for the presence of line noise, which was found to be negligible in all subjects. To evaluate the number of phases (i.e., polyphasia), the number of MEP zero-baseline crossings were measured. The number of zero (baseline) crossings of each MEP was estimated in a time window that ranged from 9 ms before to 36 ms after the first MEP peak. The length of the time window varied for each subject in order to include all possible polyphasic activity and exclude stimulus artifact. Initially, for each individual MEP of every participant, the total number of phases (zero crossings) was counted. Then, the mean number of phases was estimated over all MEPs for each participant (10–15 pulses in total). With this approach we were able to categorize the responses as polyphasic or non-polyphasic (> 2 or < 2 phases, respectively). For each participant, the ratio of the number of MEPs that contained polyphasia over the total number of MEPs that were recorded was also estimated.

### Whole Exome Sequencing

Peripheral blood samples were obtained from all study participants for genomic DNA extraction by using the Gentra Puregene Blood Kit (#158467, Qiagen, Valencia, CA, United States). DNA concentration was quantified, prior to use, with the Qubit^®^ dsDNA BR (Broad-Range) Assay Kit (Q32853, Thermo Fisher Scientific <^®^) by the Qubit 1.0 Fluorometer (Thermo Fisher Scientific <^®^). Two WES analysis approaches were applied to capture possible variants associated with polyphasia, the trio-based analysis and the two-case scenario (Figure 1 and Table 2).

#### Sequencing of 4 JME Trios Whole Exomes

The trio-based analysis was used in an effort to compare the affected individual whole exome with his/her healthy parent’s whole exome to identify any possible risk genes or variants responsible for the polyphasic activity. Four non-consanguineous families were selected for the trio-based analysis (Table 2). Trio’s included the JME proband who had the polyphasic phenotype and both of his/her parents from whom at least one of them showed polyphasia. Individual exomes of the trios were enriched using the Illumina TruSeq Exome Library Preparation kit (FC-150–1004, Illumina, San Diego, CA, United States) at CING. Fragment size of the final libraries was read by Agilent Bioanalyzer DNA (Agilent Technologies <^®^) and the 150-bp paired-end reads were sequenced on the Illumina NextSeq 500 Platform (#SY-415-1001, Illumina, San Diego, CA, United States) at CING. The raw data of the trio-based analysis are available on http://www.ebi.ac.uk/ena/data/view/PRJEB39431.

#### WES Two-Case Scenario Between Polyphasic and Non-polyphasic Cases

In the two-case scenario the whole exomes of 16 polyphasic subjects and 14 non-polyphasic were compared and analyzed to discover any genetic variants associated with polyphasia phenotype. Participants with polyphasia were three JME patients and 13 controls (16 in total), while non-polyphasic participants were one JME patient and 13 controls (14 in total). The samples data used in this analysis were obtained by the Epi25 Consortium,^1^ an international collaboration of more than 30 research groups working together (Epi25 Collaborative, 2014). These samples were provided to the Epi25 Consortium from our research cohort at CING to be sequenced as part of a collaboration agreement. Support for Epi25 data generation includes the NHGRI Centers for Common Disease Genomics (UM1HG008895). The raw data of three patients are available on dbGaP at http://www.ncbi.nlm.nih.gov/gap through dbGaP accession number phs001489 as part of the first Epi25 Collaborative publication (Epi25 Collaborative, 2019). The raw data of one patient and controls supporting the conclusions of this analysis will be made available by the authors, without undue reservation to any qualified researcher, after being published by the Epi25 Consortium as per our Collaboration agreement.

### Bioinformatics Analysis of the Genetic Data

#### Raw Data Processing, Alignment, and Variant Calling

Analysis of all data, for both trio-based and two-case scenario was performed using the Next Generation Sequencing (NGS) Analysis pipeline of the CING-Bioinformatics Group Server (C-BIG), which is an internal web platform, providing computational pipelines for in-house data analysis (for further details see Supplementary Material 1).

#### Variant Annotation

##### Trio based analysis

Variants annotation was performed using Ensembl (version 90) and tools Variant Effect Predictor (VEP, version 90.6) in order to indicate which genes and transcripts are affected by the variant and if these variants trigger functional defects. Known variants from the SNP (dbSNP) (Release 147) database were marked in order to identify novel variants with serious predicted consequences. Further data exploration was performed using Genome Mining (GEMINI, version 0.20.0), a flexible framework for exploring genome variation.

The genetic variants were filtered by the VarApp Browser. This is a reactive graphical user interface (Delafontaine et al., 2016) developed to support GEMINI and contains special filters that are applied in large sets of exome data to minimize the noise from common variants and narrow down the list of possible relevant variants. Variants can be filtered according to their quality, allele frequency, severity and pathogenicity.

Regarding the trio-based analysis, by taking into consideration the segregation of a variant in the family, each trio was examined using five possible inheritance scenarios: autosomal dominant, autosomal recessive, compound heterozygous, *de novo* and X-linked. The variants detected in the affected child were compared to the corresponding positions of the parent’s DNA. In addition, the software performs a gene-based functional annotation and recognizes the genomic region containing the variant (exonic or intronic, chromosome position, start and end position in OMIM and gene). Furthermore, a filter-based analysis is done to assess the minor allele frequency (MAF) of the variants in 1000 Genome (1000 g), Exome Aggregation Consortium (ExAC), ESP, dbSNP and to provide functional prediction scores from SIFT and PolyPhen-2 and clinical significance for each of the variant according to ClinVar. Single nucleotide polymorphisms with MAF higher than 1% in 1,000 g, ESP and ExAC were removed. The subsequent scenario-specific variants were trimmed further by including only the ones falling within coding and exonic region for at least one transcript and excluding the ones in intronic areas, or the non-functional ones. Variants were also filtered by impact, whereby, high (frameshift, start/stop lost, stop gained, splice acceptor/donor) and medium (inframe deletion/insertion, missense, protein altering and splice region) impact variants were considered for the analysis. Non-synonymous Single nucleotide Variants (SNVs) or SNVs that result in premature truncation of the encoded protein are more probable to be pathogenic.

After considering all the above limitations, a validation of the WES short-listed variants was performed comparing them with WES data of a group of 46 individuals with endocrine abnormalities related to puberty (eight males and thirty-eight females) and without any neurological disorder. Candidate variants found by WES were further validated with conventional Sanger sequencing on DNA aliquots and investigated in the rest of the patients, their relatives and the control group (Table 2).

##### Two-case scenario analysis

After obtaining the WES raw data from the Epi25 collaborative, more detailed analyses were performed in order to reveal the odds ratio and the risk score prediction of any variant is revealed and details of these analyses are described in the following sections.

###### Odds ratio analysis..

The odds ratio was calculated using PLINK, a tool containing special functions in order to obtain variants with high disease association. All data were converted into the standard PLINK format (Purcell et al., 2007). Two categories of variants were distinguished: the variants that were more frequent in polyphasic cases than non-polyphasic cases and were labeled as high-risk variants and the variants that were more frequent in non-polyphasic cases than polyphasic cases, which were labeled as low risk variants.

###### Risk score prediction using Linear Logistic Regression Analysis..

The risk score prediction was performed using the Linear Logistic Regression Analysis (LLRA) and risk model construction. PredictABEL is also used for linear logistic regression fitting in order to develop risk models assess their performance, predict risks and obtain weighted and unweighted risk scores (Kundu et al., 2011; for further details see Supplementary Material 2).

###### Machine learning data analysis..

Following the LLRA analysis, our data were divided into two classes: polyphasic (*n* = 16) and non-polyphasic (*n* = 14) to train the linear regression model classifier. In order to assess the classifier performance, the leave-one-out cross-validation (LOOCV) procedure was used (for further details see Supplementary Material 3).

###### Enrichment analysis..

Pathway-based analysis tools (Pathway Connector and EnrichR tools) were used to do an enrichment analysis by analyzing different pathways that are implicated to the phenotype and give rapidly their common relation (Chen et al., 2013; Minadakis et al., 2019; for further details see Supplementary Material 4).

### Statistical Analysis

#### Neurophysiologic Examination

Fisher’s exact test and the Chi square test were used for between-group comparison of the number of participants who had polyphasia. Statistical comparisons of the number of phases and the ratio of the number of MEPs that contained polyphasia were made. Groupwise differences were assessed with the Wilcoxon rank-sum test, followed by *post-hoc* comparison with the Kruskal-Wallis test. For all comparisons *p* < 0.05 was taken to indicate statistical significance.

#### Genetic Analysis

Fisher’s exact test and Chi square test were used to evaluate whether any polymorphism detected was significantly associated with polyphasia.

## Results

### Neurophysiological Findings

**FIGURE 2:**
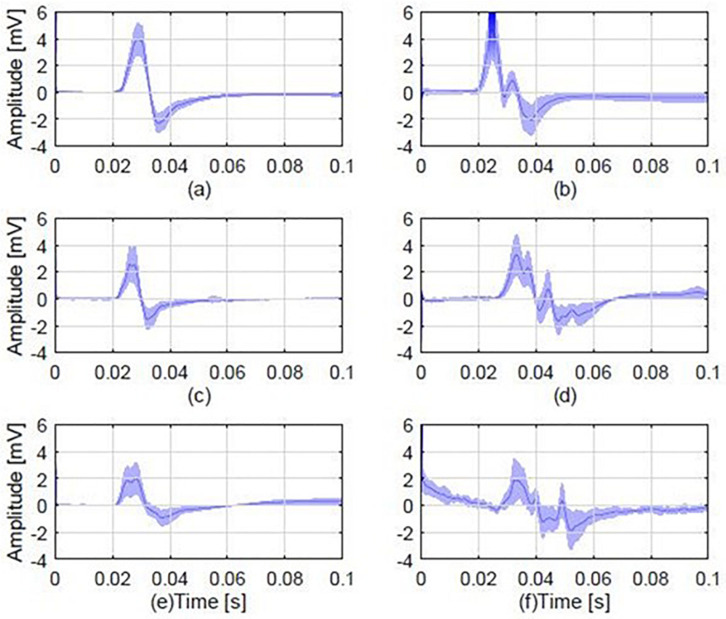
Average MEP responses from 6 different individuals, 2 from each study group, including examples of a polyphasic and a non-polyphasic recording. **(A)** Average normal MEP from a control participant; **(B)** a polyphasic MEP from a control participant with 3 phases (i.e., three zero baseline crossings); **(C)** A normal MEP from a patient; **(D)** a polyphasic MEP from a patient, displaying 4 phases; **(E)** a normal MEP obtained from the first degree relative of the patient in plot **(C)**. **(F)** A polyphasic MEP obtained from the first degree relative of the patient whose MEP is shown in **(D)**. Each plot shows the mean MEP of all stimulations (dark blue) and standard deviation (light blue shaded areas). The normal MEP shown in plot **(C)** is from a JME patient who is the parent of the healthy relative with normal MEP shown in plot **(E)**. The polyphasic MEP shown in plot **(D)** is from a JME patient who is the child of the healthy relative with the polyphasic MEP shown in plot **(F)**.

Figure 2 illustrates representative examples of mean MEP responses from six randomly selected participants from each of the three groups. Overall, 14 out of the 20 (i.e., 70%) pairs of patients-relatives that were examined for polyphasic activity presented polyphasia.

TMS-generated MEPs revealed polyphasia in more than 2/3 of the patients and relatives (17/20 ∼85%, 16/23 ∼70%, respectively), in contrast to healthy controls (14/30, ∼47%) (Table 3). This difference was statistically significant (Fisher’s Exact test: *p* = 0.018; Chi-square test: *p* = 0.014, *p* < 0.05).

**TABLE 3:** Number of participants displaying polyphasia in each group with indication of the statistical analysis performed and results obtained.

Group	Number of participants with polyphasia	Percentage (%)	Fisher’ Exact *P* < 0.05	Chi-square *P* < 0.05
Patients	17/20	85*	0.018*	0.014*
Relatives	16/23	70*		
Controls	14/30	47		

*Statistically significant.

**FIGURE 3:**
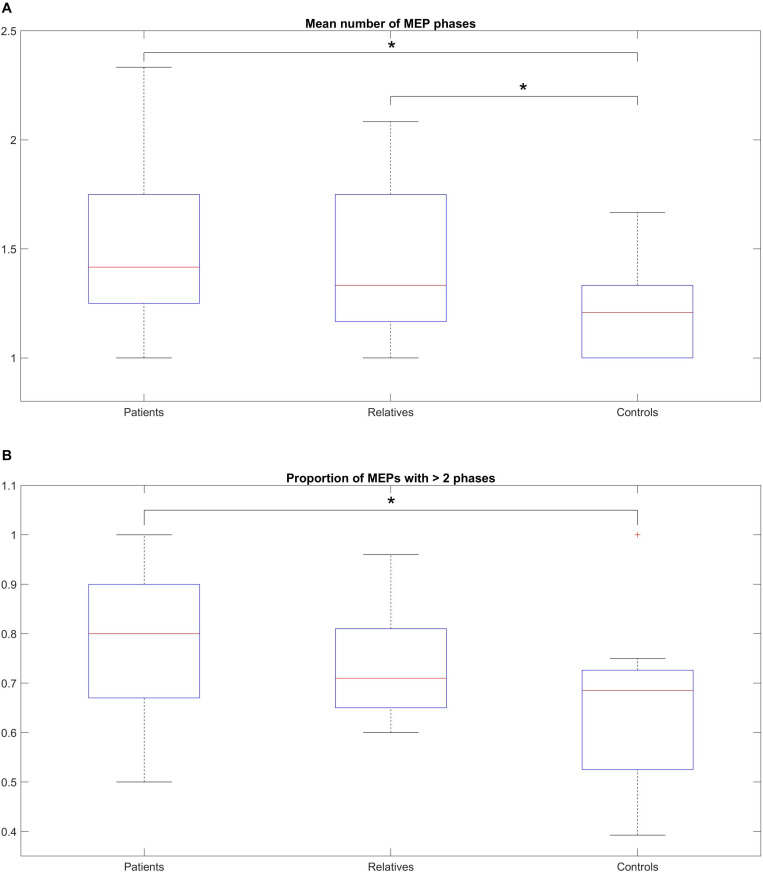
**(A)** Median number of MEP phases across the total number of stimulations, within-subjects, for patients, relatives and controls. **(B)** Proportion of MEPs with > 2 phases across the total number of stimulations, (within-subjects) for patients, relatives and controls. **(A)** The box-and-whisker plot illustrates the median, (horizontal line within the box), the 25th and 75th centiles (bottom and top of box, respectively) and the minimum and maximum values (lower and upper whiskers, respectively). Patients show a higher number of mean zero crossings compared to controls (statistically significant). The number of mean zero crossings for relatives was higher compared to controls (statistically significant). The maximum number of the mean zero crossings for each participant was also different between the groups. **(B)** The box-and-whisker plot illustrates the median, (horizontal line within the box), the 25th and 75th centiles (bottom and top of box, respectively) and the minimum and maximum values (lower and upper whiskers, respectively). A pairwise comparison of the mean ratio between patients and controls was statistically significant but no difference was observed for relatives compared to controls or patients compared to relatives.

Figure 3A shows the mean average number of zero crossings in all three groups. Patients had a higher number of mean zero crossings [mean: 1.49; range: (1–2.33); *SD*: 0.32], compared to controls [mean: 1.22; range: (1–1.66); *SD*: 0.14]. This difference was statistically significant (Wilcoxon rank-sum test, *p* = 0.003). The number of mean zero crossings for relatives [mean: 1.46; range: (1–2.08); *SD*: 0.36] was higher compared to controls, and this difference was also statistically significant (Wilcoxon rank-sum test: *p* = 0.009). The maximum number of the mean zero crossings for each participant was also different between the groups, with 2.33 for patients, 2.08 for relatives and 1.66 for controls. Details on the values are shown in Table 4A. In addition to this, a comparison of all three groups was performed displaying a significant difference (Kruskal-Wallis, *p* = 0.0001) (Table 4A). The average number of zero crossings for relatives and patients was not significantly different (Wilcoxon rank-sum test, *p* = 0.537) (Table 4A).

**TABLE 4:** Between-group statistical comparison of zero crossings (A) and proportion of MEPs (MEPs with polyphasia/total number of MEPs for a patient) (B) (group-wise comparison: Wilcoxon rank-sum test, *post-hoc* comparison: Kruskal-Wallis test).

(A) Between-group statistical comparison of zero crossings	
Groups	*P*-value (< 0.05)
Patients-relatives	0.537
Patients-controls	0.003*
Relatives-controls	0.009*
Comparing all 3 groups	0.0001*

**(B) Between-group statistical comparison of proportion of MEPs (MEPs with polyphasia/total number of MEPs for a patient)**	

**Groups**	***P*-value (< 0.05)**

Patients-relatives	0.432
Patients-controls	0.045 *
Relatives-controls	0.207
Comparing all 3 groups	0.125

*Statistically significant.

Figure 3B shows the mean ratio of the number of MEPs that contained polyphasia over the total number of MEPs that were recorded from each participant for the three groups investigated. The mean ratios were 0.77 [range: (0.5–1); *SD*: 0.15], 0.74 [range: (0.6–0.96); *SD*: 0.11] and 0.65 [range: (0.39–1); *SD*: 0.16] for patients, relatives and controls respectively. Groupwise statistical comparison did not reveal significant differences (Kruskal-Wallis, *p* = 0.125) (Table 4B). However, a pairwise comparison of the mean ratio between patients and controls was statistically significant (Wilcoxon rank-sum test, *p* = 0.045) (Table 4B). The same was not observed for relatives compared to controls (Wilcoxon rank-sum test, *p* = 0.207) or patients compared to relatives (Wilcoxon rank-sum test, *p* = 0.432) (Table 4B).

The presence of polyphasia with respect to the level of stimulation was also investigated. The highest incidence of polyphasia was observed following cortical stimulation in all groups of participants, while the lowest incidence of polyphasia was associated with spinal cord stimulation. Stimulation at both cortical and spinal levels was associated with slightly increased incidence compared to spinal stimulation alone. More specifically, out of 17 patients who had polyphasia, only 3 displayed polyphasia following spinal cord stimulation, 2 following cortical and spinal stimulation, and 12 following cortical stimulation. For the 16 relatives who displayed polyphasia, only one participant presented the examined phenotype during spinal cord stimulation and 5 during both stimulations, in contrast to 10 participants who displayed polyphasia following cortical stimulation. Finally, for the 14 healthy controls who displayed polyphasia, three participants displayed polyphasia during spinous stimulation, four during both stimulations, and seven following cortical stimulation. Details are shown on Table 5A. Despite the increased incidence of polyphasia following cortical stimulation, a Chi-square test showed that there was no significant association between presence of polyphasia and the level of stimulation (*p* > 0.05). In regards to the muscles examined, in the group of patients, 11 out of 17 had polyphasic activity on the ADM muscle, 5 on TA and one participant in both. For the group of relatives, 8 (out of 16) had polyphasic activity on ADM, 4 on TA and 4 in both muscles. Finally, in the controls group, 5 participants presented the examined phenotype on ADM examination, 6 on TA muscle and 3 on both musles. Details are shown on Table 5B. A Chi-square test did not reveal a significant difference between the type of muscle stimulated and the appearance of polyphasic activity (*p* > 0.05).

**TABLE 5:** Number of participants who presented polyphasia regarding the level of stimulation (A) and the muscles examined (B) with indication of the Chi-square statistics results (*p*-value) for both situations.

(A) Level of stimulation	Polyphasia appearance			
	Patients (*n* = 17 out of 20)	Relatives (*n* = 16 out of 23)	Controls (*n* = 14 out of 30)	Chi-square (*P* < 0.05)
Cortex	12 out of 17	10 out of 16	7 out of 14	0.495
Spinal cord	3 out of 17	1 out of 16	3 out of 14	
Both levels	2 out of 17	5 out of 16	4 out of 14	
**(B) Muscles examined**				
ADM	11 out of 17	8 out of 16	5 out of 14	0.397
TA	5 out of 17	4 out of 16	6 out of 14	
ADM and TA	1 out of 17	4 out of 16	3 out of 14	

### Genomic Trio-Based Analysis Findings

**FIGURE 4:**
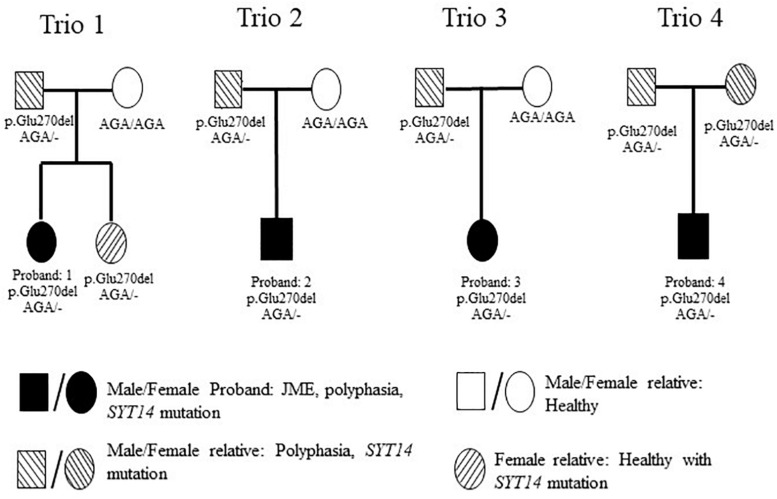
Pedigrees of the 4 investigated families with WES. Trio 1 is constituted from the JME female proband who had polyphasia and the *SYT14* mutation, her healthy sister who had the *SYT14* mutation and her parents from whom her father presented polyphasia and the *SYT14* mutation and her healthy mother without the *SYT14* mutation. Trio 2 is constituted with the JME male proband who had polyphasia and the *SYT14* mutation and his parents from whom his father presented polyphasia and the *SYT14* mutation and his healthy mother without the *SYT14* mutation. Trio 3 is constituted with the JME female proband who had polyphasia and the *SYT14* mutation and her parents from whom her father presented polyphasia and the *SYT14* mutation and her healthy mother without the *SYT14* mutation. Trio 4 is constituted with the JME male proband who had polyphasia and the *SYT14* mutation and his parents who both presented polyphasia, the *SYT14* mutation without having epilepsy.

In order to investigate possible correlations between various polymorphisms and polyphasia, WES was performed in the twelve samples of four trios (Figure 4). The quality of the sequencing of all samples examined is shown in Supplementary Table S2. The analysis was confirmed with a good coverage of the targeted regions of the sequencing with 96% average total coverage at 2X and 75% average total coverage at 20X (Supplementary Table S2).

The trio-based analysis framework established in this study allows for in-depth investigation of the polyphasic phenotype. The autosomal dominant mode of inheritance was used due to the fact that at least one parent presented polyphasia along with the affected JME proband (Figure 4). A MAF less than 1 (in 1,000 g, ESP and ExAC) was then used in order to investigate common variants found in both the patient and the parent(s) who presented polyphasic activity and were excluded from the non-polyphasic parent. The analysis revealed six variants affecting different genes (Table 6), three of which were missense with a benign-tolerated pathogenicity of low impact, while the other three variants were two splice region changes and one inframe deletion. None of these three latter variants were previously described to be implicated in epilepsy, in regards to cortical excitability and polyphasic activity. For a completed analysis, we also looked at the other modes of inheritance, but we found that no variants adhered to autosomal recessive, *de novo*, compound heterozygous and X-linked mode scenarios. Before proceeding with Sanger Sequencing validation, we checked if the identified variants are present in a control group of 46 individuals with endocrine abnormalities related to puberty. Three variants in *CCDC90B, PARP4*, and *SPYDYE1* genes were present and these were excluded from the sequencing validation.

**TABLE 6:** Candidate polymorphisms for polyphasia.

Gene	Amino acid change	Nucleotide change	Chromosome/position	Mutation impact (Medium)	MAF (ExAC)	Linked to human disorders (OMIM, ClinVar, Ensembl)
***SYT14***	**p.Glu270del**	**N/A**	**Chr1 210.267.893**	**Inframe deletion (AGA/−)**	**0.12**	**Cerebral atrophy, macrocephaly seizures and developmental delay, spinocerebellar ataxia with psychomotor retardation**
*NLRP13*	N/A	c.2109G > C	Chr19 56.426.074	Splice region	0.44	Ductal breast carcinoma, immune response to smallpox, long QT syndrome, exploratory eye movement dysfunction in schizophrenia
*SLCO4C1*	N/A	c.1878G > A	Chr5 101.575.091	Splice region	0.33	Hereditary cancer-predisposing syndrome, Type 2 diabetes, obesity
*CCDC90B*	p.Arg127Gln†	c.380C > T	Chr11 82.973.004	Missense	0.42	Intellectual disability, autism
*PARP4*	c.3794C > G	p.Gly1265Ala	Chr13 25.009.485	Missense	0.39	Cancer, sudden cardiac arrest, obesity, intracranial hypertension
*SPDYE1*	c.833T > C	p.Cys278Arg	Chr7 44.047.066	Missense	0.42	Williams-Beuren Syndrome

Among the three remaining variants, the inframe deletion, p.Glu270del, in *SYT14 (Synaptotagmin 14)* found at chromosome 1 (rs144713062 or rs2307890 at dbSNP) was considered to be a good candidate (Table 6). *SYT14* encodes the Synaptotagmin XIV protein which is part of the Synaptotagmin family proteins (Doi et al., 2011). The other two variants, *NLRP13* and *SLCO4C1*, are found to be implicated in different types of cancer, diabetes type 2, eye movement dysfunction and obesity (Table 6). *NLRP13* was not found to be expressed in the brain, so it was excluded from further consideration.

Sanger sequencing was performed only for *SYT14* variant (p. Glu270del) in all study participants (Figure 1 and Table 2). This variant was found in all individuals who presented polyphasic MEP in the trio-based analysis, either patients or relatives). The validation analysis indicated that the nine persons from the four trios who presented the polyphasic phenotype, indeed had the inframe deletion at *SYT14*. However, only seven out of 17 JME patients who presented polyphasia had the *SYT14* polymorphism (41.17%), seven out of 16 (43.75%) relatives (five from the trios) and four out of 14 (28.57%) from the group of controls. These findings are not statistically significant (Chi-Square test: *p* > 0.05) (Supplementary Table S3).

In addition, we searched online gene panels for genes that are implicated in diseases that also have polyphasia, like epilepsy, ALS and MD (Genedx, 2000; Cegat, 2009; Fulgentgenetics, 2011; Blueprintgenetics, 2012). Supplementary Table S4 shows the genes that were found to be common in these diseases. Two of them were found in two of our examined families: the Granulin Precursor (*GRN*) gene in epilepsy and ALS panels and the Pantothenate Kinase 2 (*PANK2*) gene in epilepsy and MD panels. *GRN* missense variant, p.Arg579Cys, chromosome 17, position 42,430,119 is found on our second proband and his father, who both presented polyphasia, with a medium impact change. The second missense variant, in *PANK2*, p. Ala131Gly, chromosome 20, position 3,870,139 was found only on the fourth proband, who presented polyphasia with a medium impact change.

### Genomic Two-Case Scenario Findings

**FIGURE 5:**
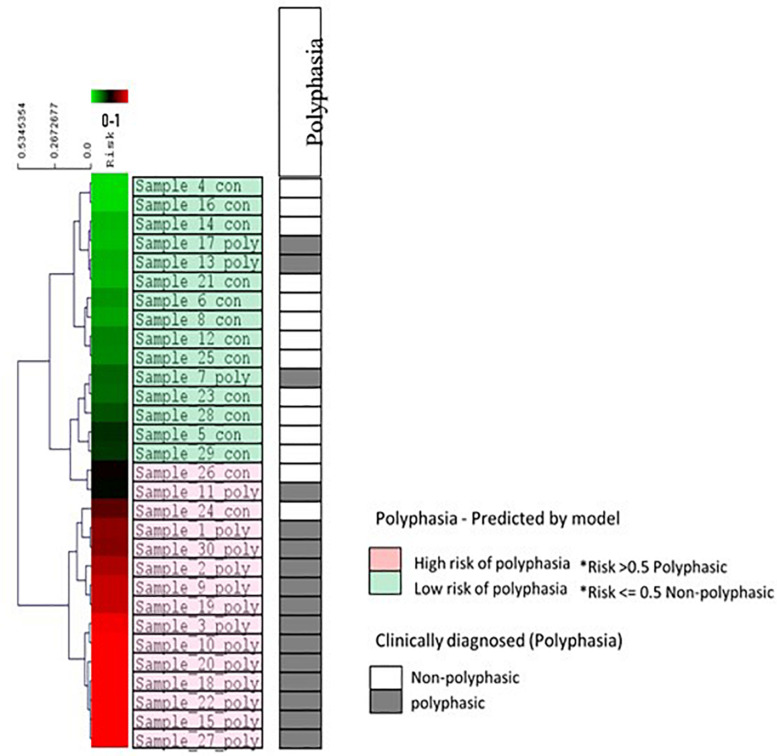
Risk prediction results for all 30 samples and other clinical data using the eight variants selected during LOOCV. The diagram indicates the hierarchical clustering of the linear regression risk prediction outputs (Euclidean distance, average linkage algorithm). The visualization shows results from the propagation of all samples through the trained model. A complete spectrum of risk prediction scores is illustrated in the green/red gradient running along the dendrogram, sorted by severity in descending order. Color coding is according to the molecular subtypes as predicted by the risk models (light green color boxes are the low-risk cases for polyphasia and the pink color boxes are the high-risk cases for polyphasia). The polyphasia column, shows the clinical diagnosis of polyphasia, whereby the gray boxes show the cases with polyphasia and the white boxes the non-polyphasic cases.

A machine learning approach was used to find possible variants that could be associated with polyphasia and to estimate a risk score for polyphasia appearance in the two groups examined (polyphasic, *n* = 16, and non-polyphasic, *n* = 14) (Figure 1 and Table 2). The optimal set of variants was coupled to the performance to assess the classifier LOOCV procedure. The top 16 variants for every LOOCV repetition were used for the downstream pathway analysis (see details in Supplementary Figure S1). In order to evaluate the performance of every feature selection run, four parameters were assessed: the prediction accuracy, specificity, sensitivity and Mathew’s Correlation Coefficient (MCC), as shown in Supplementary Figure S2. Overall, the optimum results obtained by our risk model-defined molecular subtypes were in agreement with prior clinical grading with 83.33% (25/30 samples) prediction accuracy, 81.25% sensitivity, 85.71% specificity and 0.67 MCC. Supplementary Figure S3 illustrates the receiver operating characteristic (ROC) curves that resulted in an Area Under the Curve (AUC) of 0.87. The risk model outputs were further assessed using clustering algorithms for all samples, as shown in Figure 5. The risk prediction results revealed that from the 14 non-polyphasic subjects, 12 obtained lower risk of polyphasic activity while the two “misclassified” control samples (Sample_24 and Sample_26) were at borderline cases between low- and high-risk scores (Figure 5). Proceeding with the polyphasic samples, 13 of them showed high risk scores and only three of them showed low risk for polyphasic activity, with one of them also being borderline case (Sample_7) (Figure 5).

The top 16 variants taken from every LOOCV repetition were pooled together and a total of 61 unique variants were obtained (Supplementary Table S5 and Supplementary Figure S4). In order to narrow down the list we focused on variants present within genes that are expressed in the brain. The number of variants was reduced to 18 (Supplementary Table S6) and this list was further investigated via bibliographic research. Two of them were found to be expressed in the cerebral cortex but only one was implicated in epilepsy. This was an intronic variant (rs2248440) in the 5-Hydroxytryptamine Receptor 2C (*HTR2C)*. *HTR2C* gene plays a role in the signaling of the serotonin neurotransmitter and modulates neuronal activity in the thalamocortical loop that is the main network for absence seizures generation (Isaac, 2005; Panczyk et al., 2015).

### Pathway Analysis

Following the uncovering of these 18 variants, pathway analysis was performed in order to investigate their possible involvement in common pathways. The Pathway Connector as well as the EnrichR online tools were used for this assessment (Chen et al., 2013; Kuleshov et al., 2016; Minadakis et al., 2019). The list of genes found to be statistically significant (*p* < 0.05) in sharing common pathways in both tools is shown in Supplementary Table S7. Supplementary Figure S5 provides data on the top gene-to-pathway (Supplementary Figure S5A) and pathway-to-pathway interactions (Supplementary Figure S5B), with potential implications and associations with the appearance of polyphasic activity. The pathways revealed are implicated in various metabolic paths such as Histidine metabolism, bile secretion, cancer such as colorectal cancer, ribosome formation, and one carbon pool by folate. In the enrichment analysis *HTR2C* gene was found to be implicated with Serotonin receptors-2 and Elk-SRF/GATA4 signaling.

## Discussion

This is the first detailed study that (1) examines polyphasic MEPs evoked by TMS in JME, comparing them with their healthy first-degree relatives and a group of controls, and (2) investigates the possible genetic architecture that could be responsible for this cortical pathophysiology.

The present study revealed increased MEP polyphasia induced by TMS in the group of JME patients (85%) and their healthy relatives (70%) compared to controls (45%). Polyphasia was present in 14 out of the 20 pairs of patients-relatives. In addition, we observed that the mean number of MEP zero crossings in both patients and relatives was significantly higher (1.49 and 1.46, respectively) than controls (1.22). For the proportion of MEPs with more than two phases, the mean number was significantly higher in patients than in controls (0.77 and 0.65, respectively).

Epilepsy is characterized by over-excitability of neurons and rise of synchronicity of the neuronal networks, as a result of an imbalance between excitatory and inhibitory neurotransmitters in the brain (Engelborghs et al., 2000). TMS can provide information on the status of cortical excitability and its underlying mechanisms. Assessments done with TMS rely on the excitations and inhibitions that arise from interneuronal networks, synapses between them and motor neurons (Rothwell, 1997). TMS has shown that patients with different types of epilepsies have an increased level of cortical excitability (Manganotti et al., 2000; Badawy et al., 2007). JME patients’ cortical inhibition is deficient more so in the morning, which would explain the sensitivity these patients have upon awakening. However, the mechanism responsible for the polyphasic MEP remains unknown. It has been suggested that a probable temporal dispersion of the fast monosynaptic descending volleys may be the underlying cause of a more complicated MEP waveform, and that polysynaptic or decelerated monosynaptic pathways can cause alterations to the MEP (Kohara et al., 1999). Changes in the time of discharging and recruitment of the spinal descending motor neurons may result in a polyphasic MEP (Van Der Salm et al., 2009). In other studies, examining ALS, MD and different types of IGE, researchers assigned this phenomenon of polyphasia to asynchronous oscillations and to central mechanisms with the participation of corticospinal pathways (Kohara et al., 1999; Van Der Salm et al., 2009). Thus, it is likely that the same mechanism also underlies the polyphasia observed in this study.

Epilepsy depends on alterations in the activity of ion-channels leading to either hyperexcitability or inhibition (Oyrer et al., 2018). Malfunction of excitatory channels, such as Hyperpolarization-Activated Cyclic Nucleotide-Gated channels (HCN) and an intracortical inhibition because of an impaired GABA mediated mechanism (GABA-A) (Yu et al., 2006), with an extra stimulation of the motor cortex with TMS will result in more intense waves that will spread around the neuronal networks. This abnormal firing of waves down the corticospinal tract will result in a non-synchronized fluctuation in the already dysfunctional circuits, giving rise to a polyphasic MEP.

Besides patients, their healthy relatives also exhibited polyphasia. It is worth mentioning that only the group of JME patients was on antiepileptic medication (AED). Some AEDs, such as valproic acid (VPA) and lamotrigine (LTG), can inhibit voltage-gated sodium channels that are responsible for excitation, however, VPA can also increase the GABA-mediated neurotransmission responsible for the inhibition. A number of TMS studies indicate that differences in some measurements of MEPs between JME patients and controls, such as motor threshold cortical silence period central motor conduction time and resting motor threshold can be attributed to the different types of epilepsy and AEDs (Akgun et al., 2009). In addition, studies show differences in MEP measurements of patients before and after anticonvulsant treatment (Reutens et al., 1993). In general, many studies suggest that medication may play a role in the differences between patients and controls proposing that this may act as a protective mechanism against seizures (Delvaux et al., 2001; Varrasi et al., 2004; Kotova and Vorob’eva, 2007). The fact that the healthy untreated first-degree relatives had polyphasia cannot be attributed to the effect of AEDs. Thus, it is likely that patients and relatives share a faulty mechanism of cortical inhibition or hyper-excitability that is asymptomatic in relatives (Badawy et al., 2013), which in turn could be due to a common, abnormal genetic mechanism. Therefore, we also investigated the hypothesis that the specific cortical characteristic of polyphasia, which is present in both JME patients and their healthy first-degree relatives, is linked to a common genetic trait that, on its own, may not result in the phenotype of JME. To our knowledge, this is the first time this hypothesis was investigated with WES analysis approaches in JME patients, their relatives and healthy controls in order to correlate the polyphasic activity with possible genetic variants. Known that epilepsy alone has a complicated and challenging genetic background, along with the newly appeared and complex polyphasic trait, the expectation on discovering specific variants associated with the phenotype is not overrated.

The trios-based WES analysis revealed a possible polymorphism in *SYT14* gene for the appearance of polyphasia. All individuals who participated in the trio-based analysis and also presented polyphasia showed the inframe deletion, p. Glu270del, on *SYT14* and this was further validated with Sanger sequencing. *SYT14* gene encodes for a membrane protein, Synaptotagmin XIV, which is mostly found to be linked with the process of exocytosis in secretory vesicles, comprising the synaptic vesicles that are implicated in neuronal synapses (Doi et al., 2011). Quintero-Rivera and colleagues have demonstrated that a *de novo* balanced translocation t (1;3) (q32.1; q25.1) found in a 12-year old female suffering with cerebral atrophy, developmental delay and absence seizures can alter one of the *SYT14* alleles, contributing to the neurodevelopmental abnormalities seen. Since *SYT14* is implicated in absence seizures, these genetic findings can be related with the JME phenotype, which is characterized by the existence of absence seizures. Two of our four JME probands in the trio analysis (proband 1 and proband 4) appear to have absence seizures in a weekly-monthly basis. However, the Sanger validation showed that in the rest of our investigated JME patients, relatives and controls, not all who had polyphasia presented this alteration of the gene (see details in Results 3.2). As a conclusion, these results cannot be associated with our electromyography phenotype since the *SYT14* variant was not significantly observed in the participants with polyphasic activity.

The shared genes between ALS, MD, and epilepsy, diseases presenting polyphasia, were examined in our trios for the presence of any genetic variants. Two of them were found in two families, separately, the *GRN* gene and the *PANK2* gene. *GRN* missense variant, p. Arg579Cys, was found on our second proband and his father, both of whom had polyphasia. This variant is being referred as deleterious and is implicated in generalized seizures and ceroid lipofuscinosis (NCL). In NCL, myoclonic epilepsy is a common symptom of the disease (Chabrol et al., 2013; Nita et al., 2016). The second missense variant found in *PANK2* (p. Ala131Gly), was present in the fourth proband, who had polyphasia. It is described as deleterious with low confidence and it is associated with neurodegeneration with brain iron accumulation 1 with a rare appearance of seizures. No correlation was found with *GRN* or *PANK2* and JME or polyphasic activity.

Regarding the two-case scenario analysis, 61 variants were revealed from the bioinformatics investigation. After an extensive bibliographical search to check whether these genes have any connection with the examined phenotype or epilepsy, one intronic variant (rs2248440) was revealed, in *HTR2C*. HTR2C is expressed in the central nervous system and, in addition to its implication in serotonin signaling and neuronal excitability, it is also involved in neuropsychiatric disorders, mental and behavioral disorders (Felsing et al., 2018). Recently, there has been some evidence of the involvement of the serotonergic system (including different aliases of *HTR2C* such us 5-*HT1A, 5-HT2C, 5-HT3*) in the pathomechanism of epilepsy and its contribution on provoking spontaneous and recurring seizures either by increasing or decreasing neuronal excitability (Isaac, 2005; Panczyk et al., 2015). More studies on these genes could enlighten their function and their relation to polyphasia. Our study did not detect variants associated directly with polyphasic activity, however, sets of variants in different genes combined may increase the risk of polyphasia presence. The risk prediction scores revealed that our non-polyphasic subjects (subjects who did not have polyphasia with TMS examination) obtained a lower risk for polyphasic appearance, while our polyphasic subjects (subjects who had polyphasia with TMS examination) had an increase risk on having polyphasia. Therefore, the risk prediction scores results are indeed in agreement with our TMS results (i.e., subjects that presented polyphasia and subjects that did not present polyphasia). The pathway enrichment analysis revealed that many of the investigated genes share common mechanisms and are involved in similar biological processes but are not directly functionally linked with polyphasia and/or epilepsy.

In this study we focused on neurophysiological and genetic findings in patients with JME phenotype. This has not been done before distinctively in this type of GGE. Our findings show that polyphasia is significantly present in both groups of patients and relatives compared to controls. This is in concordance with a previous study performed in patients with various of GGE syndromes. To the best of our knowledge, there are no known clinical symptoms that are linked to polyphasia which can give us a clearer clinical image of the patients. We did not find any direct association between the genetic variants obtained and polyphasia. However, these findings can contribute toward improving our understanding of polyphasia cause and its relation to epilepsy, ALS, MD and other diseases presenting polyphasia. Future work that investigates more types of GGE and larger cohorts may provide a clearer picture on the examined characteristic, illustrating that it may be exclusive to specific GGE types. This may help reveal any association between polyphasic activity and risk genes/variants.

## Data Availability Statement

The original contributions presented in the study are publicly available. This data can be found here: http://www.ebi.ac.uk/ena/data/view/PRJEB39431.

## Ethics Statement

The studies involving human participants were reviewed and approved by the Cyprus National Bioethics Committee (EEBK/EΠ/2016/33). The patients/participants provided their written informed consent to participate in this study.

## Author Contributions

SS contributed to the acquisition, analysis, and interpretation of data for the work, and wrote the first draft of the manuscript. SP contributed to the conception and design of the work, recruited the participants, and provided the approval for publication of the content. IK helped with the genetic experiments. IK and SP supervised the study. NN and EP helped with the neurophysiological analysis. AO and GS helped with the bioinformatics analysis. PF helped with the genetic analysis. PF, VN, and LP provided some clinical data for the project. SS, IK, NN, EP, GS, and SP contributed to the manuscript revision, read and approved the submitted version. All authors contributed to the article and approved the submitted version.

## Conflict of Interest

The authors declare that the research was conducted in the absence of any commercial or financial relationships that could be construed as a potential conflict of interest.

## Footnotes

1 www.epi-25.org

## Abbreviations

ADM: Abductor Digiti Minimi
AED: antiepileptic drugs
ALS: Amyotrophic Lateral Sclerosis
dbSNP: data base SNP
EEG: Electroencephalogram
ExAC: Exome Aggregation Consortium
Gemini: Genome Mining
GGE: Generalized Genetic Epilepsy
*GRN*: Granulin Precursor
*HTR2C*: 5-Hydroxytryptamine Receptor 2C
IGE: Idiopathic Generalized Epilepsy
JME: Juvenile Myoclonic Epilepsy
LLRA: Linear Logistic Regression Analysis
LOOCV: leave-one-out Cross-validation
MCC: Matthew’s correlation coefficient
MEP: Motor Evoked Potential
MD: Myoclonus Dystonia
*PANK2*: Pantothenate Kinase 2
*RPL13*: Ribosomal Protein L13
*SYT14*: Synaptotagmin 14
TA: Tibialis Anterior
TMS: Transcranial Magnetic Stimulation
WES: Whole Exome Sequencing.
