# Supplementary material for: Neurophysiological and Genetic Findings in Patients With Juvenile Myoclonic Epilepsy
Source: Front Integr Neurosci. 2020 Aug 20;14:45. doi: 10.3389/fnint.2020.00045 (PMC7468511; doi:10.3389/fnint.2020.00045)
Supplement: Supplementary file 1 [file Table_1.pdf]

Supplementary Table 1: First-degree relatives and controls age range.

	Age	
	**Relatives**	**Controls**
1	**50-55**	**30-35**
**2**	**40-45**	**25-30**
**3**	**50-55**	**25-30**
**4**	**55-60**	**30-35**
**5**	**40-45**	**25-30**
**6**	**40-45**	**35-40**
**7**	**20-25**	**25-30**
**8**	**55-60**	**25-30**
**9**	**20-25**	**25-30**
**10**	**60-65**	**25-30**
**11**	**60-65**	**30-35**
**12**	**20-25**	**35-40**
**13**	**50-55**	**25-30**
**14**	**55-60**	**30-35**
**15**	**25-30**	**25-30**
**16**	**45-50**	**25-30**
**17**	**55-60**	**25-30**
**18**	**60-65**	**30-35**
**19**	**30-35**	**25-30**
**20**	**60-65**	**25-30**
**21**	**40-45**	**45-50**
**22**	**20-25**	**25-30**
**23**	**25-30**	**45-50**

**24**		**25-30**
**25**		**25-30**
**26**		**45-30**
**27**		**20-25**
**28**		**35-40**
**29**		**40-45**
**30**		**40-45**
