# Supplementary material for: Neurophysiological and Genetic Findings in Patients With Juvenile Myoclonic Epilepsy
Source: Front Integr Neurosci. 2020 Aug 20;14:45. doi: 10.3389/fnint.2020.00045 (PMC7468511; doi:10.3389/fnint.2020.00045)
Supplement: Supplementary file 2 [file Table_2.pdf]

<u>Supplementary Table S2</u>: Details on the sequencing depth and coverage for each individual of each trio.

Trio	Individual	Coverage at 2X	Coverage at 10x	Coverage at 20x	Coverage at 30x	Coverage at 50x	Coverage at 100x
**1**	Pr	94.76%	86.96%	71.84%	54.26%	26.28%	3.14%
	F	95.44%	87.48%	71.22%	51.55%	22.34%	1.85%
	M	95.82%	90.24%	78.22%	61.67%	31.11%	4.14%
**2**	Pr	95.73%	89.24%	74.96%	55.82%	24.81%	2.27%
	F	95.98%	90.53%	77.96%	60.66%	29.94%	3.63%
	M	95.59%	89.69%	77.57%	60.45%	28.78%	3.24%
**3**	Pr	95.53%	88.76%	74.41%	55.51%	25.11%	2.26%
	F	95.75%	89.21%	74.97%	56.17%	25.44%	2.41%
	M	95.61%	88.64%	73.61%	53.68%	23.02%	1.65%
**4**	Pr	95.94%	90.68%	79.59%	64.32%	34.00 %	5.49%
	F	95.60%	87.81%	71.29%	50.92%	21.30%	1.46%
	M	95.30%	86.42%	65.17%	40.55%	13.53%	0.41%
**Average**		95.59%	88.81%	74.23%	55.46%	25.47%	2.66%

Notes:
Abbreviations: F: Father, M: Mother, Pr: Proband
