# Supplementary material for: Neurophysiological and Genetic Findings in Patients With Juvenile Myoclonic Epilepsy
Source: Front Integr Neurosci. 2020 Aug 20;14:45. doi: 10.3389/fnint.2020.00045 (PMC7468511; doi:10.3389/fnint.2020.00045)
Supplement: Supplementary file 3 [file Table_3.pdf]

**<u>Supplementary Table S3:</u>** Sanger sequencing validation for the candidate gene *SYT14*.

| Candidate Gene *SYT14* | | | | |
|---|---|---|---|---|
| **Groups** | **Individuals with polyphasia** | **Individuals with polyphasia and SYT14 mutation** | **Percentage (%) of individuals with polyphasia and SYT14 mutation** | **Significance** |
| Patients | 17/20 | 7/17 | 41.17 | p>0.05 |
| Relatives | 16/22 | 7/16 | 43,75 | p>0.05 |
| Controls | 14/30 | 4/14 | 28.57 | p>0.05 |
