# Supplementary material for: Neurophysiological and Genetic Findings in Patients With Juvenile Myoclonic Epilepsy
Source: Front Integr Neurosci. 2020 Aug 20;14:45. doi: 10.3389/fnint.2020.00045 (PMC7468511; doi:10.3389/fnint.2020.00045)
Supplement: Supplementary file 4 [file Table_4.pdf]

Supplementary Table S4: Common genes reported in Epilepsy, ALS and MD gene panels.

Comparison of genes in different disorders			
**Myoclonus Dystonia-Epilepsy**		**ALS-Epilepsy**	
**Genes**	**Type of epilepsy found**	**Genes**	**Type of epilepsy found**
ACTB	beyond pediatric epilepsy	ABCD1	beyond pediatric epilepsy, comprehensive epilepsy
ANO3	epileptic encephalopathy	GBE1	epilepsy, metabolic and brain developmental disorders
ARSA	adolescent & adult epilepsy, childhood epilepsy, neonatal epilepsy	**GRN**	**adolescent & adult epilepsy, comprehensive epilepsy, progressive myoclonic epilepsy**
ATP1A3	infantile epilepsy, epilepsy deletion/duplication, childhood onset	HNRNPA1	beyond pediatric epilepsy
AUH	Childhood epilepsy, neonatal epilepsy	HEXA	neonatal, childhood, adolescent & adult epilepsy, comprehensive epilepsy
BCAP31	beyond pediatric epilepsy	HSPD1	comprehensive epilepsy, beyond pediatric epilepsy
DDC	comprehensive epilepsy	KIF5A	epilepsy, metabolic and brain development disorders
DNAJC12	epilepsy, metabolic and brain development disorders	NEFH	beyond pediatric epilepsy
FA2H	comprehensive epilepsy, metabolic and brain development disorders	TARDBP	beyond pediatric epilepsy
GCDH	Neonatal epilepsy, comprehensive epilepsy		
GCH1	comprehensive epilepsy		
HEXA	Neonatal epilepsy, childhood epilepsy, adolescent & adult epilepsy		

KCNMA1	familial and idiopathic epilepsy, infantile epilepsy, childhood onset epilepsy, neonatal epilepsy
MECP2	epileptic encephalopathy, infantile epilepsy, stat epilepsy, childhood onset epilepsy, neonatal epilepsy
NPC1	adolescent-adult epilepsy, childhood epilepsy, neonatal epilepsy
NPC2	adolescent & adult epilepsy, childhood epilepsy, neonatal epilepsy
**PANK2**	**adolescent & adult epilepsy, childhood epilepsy**
PLA2G6	Childhood epilepsy, neonatal epilepsy
PRRT2	familial and idiopathic epilepsy, infantile epilepsy, deletion/duplication epilepsy, neonatal epilepsy
SGCE	Juvenile myoclonic epilepsy
SLC2A1	idiopathic generalized epilepsy, infantile epilepsy, familial and idiopathic epilepsy, childhood onset epilepsy, epileptic encephalopathy, neonatal epilepsy, stat epilepsy
SMPD1	Epilepsy, metabolic and brain development disorders
TAF1	comprehensive epilepsy
