# Supplementary material for: Neurophysiological and Genetic Findings in Patients With Juvenile Myoclonic Epilepsy
Source: Front Integr Neurosci. 2020 Aug 20;14:45. doi: 10.3389/fnint.2020.00045 (PMC7468511; doi:10.3389/fnint.2020.00045)
Supplement: Supplementary file 5 [file Table_5.pdf]

Supplementary Table S5: Candidate variants found for polyphasic vs non-polyphasic molecular classification selected from LOOCV.

Gene	Rs number	Chromosome	Amino acid change	Impact	ClinVar Disease	ClinVar Phenotype	Expressed in Nervous system/Brain
ECE1	rs12562197	chr1	N/A	Splice region	N/A	Hirschsprung disease, cardiac defects and autonomic dysfunction	N/A
ZSWIM5	rs4454584	chr1	A	synonymous	N/A	N/A	N/A
KNCN	rs10890404	chr1	N/A	3 prime UTR	N/A	N/A	Hypothalamus, basal ganglia, nucleus accumbens
APOB	rs1367117	chr2	T/I	missense	Familial hypercholesterolemia, familial hypobetalipoproteinemia	Familial hypercholesterolemia/hypobetalipoproteinemia autosomal dominant, normotriglyceridemic	N/A
MSH2	rs3732183	chr2	N/A	intron	Lynch syndrome, Hereditary cancer	Colorectal cancer, nonpolyposis, hereditary cancer predisposing syndrome, lynch syndrome, msh2 polymorphism, muir-torr syndrome, ovarian cancer, renal cell carcinoma tumor susceptibility linked to germline bap1 mutations, turcot syndrome	N/A
FAM161A	rs62148138	chr2	N/A	3 prime UTR	Retinitis Pigmentosa, Recessive	Retinal dystrophy, retinitis pigmentosa recessive 28	Neurons (rod cells)
FAM161A	rs146249980	chr2	N/A	3 prime UTR	Retinitis Pigmentosa, Recessive	Retinal dystrophy, retinitis pigmentosa recessive 28	Neurons (rod cells)
FAM161A	rs17513722	chr2	I/V	missense	Retinitis Pigmentosa, Recessive	Retinal dystrophy, retinitis pigmentosa recessive 28	Neurons (rod cells)
FAM161A	rs62149863	chr2	N/A	intron	N/A	Retinal dystrophy, retinitis pigmentosa recessive 28	Neurons (rod cells)
MUC7	rs6826961	chr4	N/A	missense	N/A	N/A	Cerebellum
SLC9A3	rs2230437	chr5	A	synonymous	N/A	Diarrhea, secretory sodium, congenital	N/A

ZDHHC11	rs1809008	chr5	R/Q	missense	N/A	N/A	N/A
C7orf25	rs647117	chr7	P/L	missense	N/A	N/A	N/A
PSMA2	rs55912267	chr7	N/A	intron	N/A	N/A	N/A
MRPL32	rs630191	chr7	N/A	intron	N/A	N/A	N/A
MRPL32	rs631132	chr7	T	synonymous	N/A	N/A	N/A
POR	rs1057870	chr7	S	synonymous	Antley-Bixier Syndrome with genital anomalies and steroidogenesis	Antley-Bixier syndrome with genital anomalies and disordered steroidogenesis due to cytochrome p450 oxidoreductase deficiency	N/A
TRIM56	rs4524722	chr7	A	synonymous	N/A	N/A	N/A
TRIM56	rs6948536	chr7	A	synonymous	N/A	N/A	N/A
TNKS	rs7006985	chr8	T	synonymous	N/A	N/A	N/A
WRN	rs1346044	chr8	C/R	missense	Werner Syndrome	Werner Syndrome	N/A
EYA1	rs3779747	chr8	N/A	intron	N/A	Anterior segment anomalies and cataract, branchiootic syndrome, branchiootorenal syndrome, malignant tumor of prostate, melnick-fraser syndrome, otofaciocervical syndrome, renal hypoplasia	N/A
PAEP	rs783774	chr9	N/A	intron	N/A	N/A	N/A
ADAMTS14	rs10999516	chr10	S/N	missense	N/A	N/A	Nerve-Tibial
ADAMTS14	rs4747096	chr10	E/G	missense	N/A	N/A	Nerve-Tibial
CDH23	rs1227049	chr10	G/A	missense	Retinitis pigmentosa, deafness syndrome, non-syndromic hearing loss	Atypical Gaucher disease, deafness, galactosylceramide beta-galactosidase deficiency, combined saposin deficiency, inborn genetic disease, metachromatic leukodystrophy, nonsyndromic hearing loss, retinal dystrophy, retinitis pigmentosa deafness syndrome, usher syndrome.	Hypothalamus
ARMS2	rs10490924	chr10	A/S	missense	Age related Macular degeneration	Age related Macular degeneration 8	N/A
RAB3IL1	rs174473	chr11	T	synonymous	N/A	N/A	N/A

RAB3IL1	rs174474	chr11	R	synonymous	N/A	N/A	N/A
CHORDC1	rs12786959	chr11	N/A	intron	N/A	N/A	N/A
CHORDC1	rs12787117	chr11	N/A	intron	N/A	N/A	N/A
B9D1	rs7221299	chr17	N/A	intron	N/A	Familial aplasia of the vermis Joubert syndrome 27 and related disorders, Meckel-gruber syndrome	N/A
AXIN2	rs7219582	chr17	N/A	intron	N/A	Carcinoma of colon, hereditary cancer-predisposing syndrome, non-syndromic oligodontia, oligodontia-colorectal cancer syndrome	N/A
AXIN2	rs35399989	chr17	N/A	intron	N/A	Carcinoma of colon, hereditary cancer-predisposing syndrome, non-syndromic oligodontia, oligodontia-colorectal cancer syndrome	N/A
IGSF9B	rs10894768	chr11	P	synonymous	N/A	N/A	Cerebellum
KLRF2	rs1797517	chr12	V/I	missense	N/A	N/A	N/A
SLCO1B3	rs60140950	chr12	G/A	missense	Rotor Syndrome	Rotor syndrome	N/A
KRT73	rs61736102	chr12	N	synonymous	N/A	N/A	N/A
LATS2	rs2770928	chr13	G/S	missense	N/A	N/A	N/A
LATS2	rs558614	chr13	A/V	missense	N/A	N/A	N/A
VASN	rs3810818	chr16	E/A	missense	N/A	N/A	Neurons (nGnG Amacrine cells)
C16orf62	rs957676	chr16	D	synonymous	N/A	N/A	Nervous System
USP31	rs10083789	chr16	R/L	missense	N/A	N/A	N/A
ERN2	rs26764	chr16	S/T	missense	N/A	N/A	N/A
RPL13	rs9930567	chr16	A/T	missense	N/A	Spastic paraplegia 7, recessive	Cerebral Cortex
ASPA	rs12948217	chr17	Y	synonymous	Spongy degeneration of CNS	Canavan disease, palmoplantar keratoderma, mutilating with periorificial keratotic plaques, spongy degeneration of the CNS	Hippocampus, substantia nigra
PRODH2	rs12462360	chr19	P	synonymous	N/A	N/A	N/A
PRODH2	rs3848666	chr19	P/R	missense	N/A	N/A	N/A

ZNF571	rs8111790	chr19	Q/H	missense	N/A	N/A	N/A
WDR87	rs73027454	chr19	F	synonymous	N/A	N/A	N/A
ATRN	rs151519	chr20	Y	synonymous	N/A	N/A	N/A
MCM8	rs236110	chr20	Q/K	missense	N/A	Premature ovarian failure 10	N/A
RBM11	rs378280	chr21	N/A	Splice region	N/A	N/A	Neural Tube
FTCD	rs1047209	chr21	S	synonymous	N/A	Collagen vi-related myopathy, glutamate formiminotransferase deficiency, myosclerosis	Medulla, Midbrain
PISD	rs9956	chr22		3'UTR	N/A	Childhood-onset schizophrenia	Thalamus, Cerebellum, Cerebral Cortex
FAM109B	rs1807493	chr22	A/G	missense	N/A	N/A	N/A
SLC9A7	rs1056846	chrX	A	synonymous	N/A	N/A	Nucleus Accumbens
HDX	rs35161124	chrX	F/S	missense	N/A	N/A	N/A
GUCY2F	rs502209	chrX	R/Q	missense	N/A	N/A	N/A
HTR2C	rs2248440	chrX	N/A	intron	N/A	Malignant tumor of prostate serotonin 5-ht-2c receptor polymorphism antipsychotics response toxicity, clozapine response toxicity, olanzapine response toxicity, risperidone response	Brain
UPF3B	rs2428212	chrX	N/A	intron	N/A	Mental retardation, x2c syndromic 14, x2c x-linked, non-syndromic x-linked intellectual disability	Cerebellum, Lateral Ventricle

Note: CNS: central nervous system, LOOCV: leave-one-out cross-validation, N/A: non-available
