# Supplementary material for: Neurophysiological and Genetic Findings in Patients With Juvenile Myoclonic Epilepsy
Source: Front Integr Neurosci. 2020 Aug 20;14:45. doi: 10.3389/fnint.2020.00045 (PMC7468511; doi:10.3389/fnint.2020.00045)
Supplement: Supplementary file 6 [file Table_6.pdf]

Supplementary Table S6: Candidate variants of polyphasic vs non-polyphasic molecular classification.

Gene	Rs number	Chromosome	Amino acid change	Impact	ClinVar Disease	ClinVar Phenotype	Expressed in Nervous system/Brain
KNCN	rs10890404	chr1	N/A	3 prime UTR	N/A	N/A	Hypothalamus, basal ganglia, nucleus accumbens
FAM161A	rs62148138	chr2	N/A	3 prime UTR	Retinitis Pigmentosa, Recessive	Retinal dystrophy, retinitis pigmentosa recessive 28	Neurons (rod cells)
FAM161A	rs146249980	chr2	N/A	3 prime UTR	Retinitis Pigmentosa, Recessive	Retinal dystrophy, retinitis pigmentosa recessive 28	Neurons (rod cells)
FAM161A	rs17513722	chr2	I/V	missense	Retinitis Pigmentosa, Recessive	Retinal dystrophy, retinitis pigmentosa recessive 28	Neurons (rod cells)
FAM161A	rs62149863	chr2	N/A	intron	N/A	Retinal dystrophy, retinitis pigmentosa recessive 28	Neurons (rod cells)
MUC7	rs6826961	chr4	N/A	missense	N/A	N/A	Cerebellum
CDH23	rs1227049	chr10	G/A	missense	Retinitis pigmentosa, deafness syndrome, non-syndromic hearing loss	Atypical Gaucher disease, deafness, galactosylceramide beta-galactosidase deficiency, combined saposin deficiency, inborn genetic disease, metachromatic leukodystrophy, nonsyndromic hearing loss, retinal dystrophy, retinitis pigmentosa deafness syndrome, usher syndrome.	Hypothalamus
IGSF9B	rs10894768	chr11	P	synonymous	N/A	N/A	Cerebellum
VASN	rs3810818	chr16	E/A	missense	N/A	N/A	Neurons (nGnG Amacrine cells)
C16orf62	rs957676	chr16	D	synonymous	N/A	N/A	Nervous System
RPL13	rs9930567	chr16	A/T	missense	N/A	Spastic paraplegia 7, recessive	Cerebral Cortex
ASPA	rs12948217	chr17	Y	synonymous	Spongy degeneration of CNS	Canavan disease, palmoplantar keratoderma, mutilating with periorificial keratotic plaques, spongy degeneration of the CNS	Hippocampus, substantia nigra

RBM11	rs378280	chr21	N/A	Splice region	N/A	N/A	Neural Tube
FTCD	rs1047209	chr21	S	synonymous	N/A	Collagen vi-related myopathy, glutamate formiminotransferase deficiency, myosclerosis	Medulla, Midbrain
PISD	rs9956	chr22		3’UTR	N/A	Childhood-onset schizophrenia	Thalamus, Cerebellum, Cerebral Cortex
SLC9A7	rs1056846	chrX	A	synonymous	N/A	N/A	Nucleus Accumbens
HTR2C	rs2248440	chrX	N/A	intron	N/A	Malignant tumor of prostate serotonin 5-ht-2c receptor polymorphism antipsychotics response toxicity, clozapine response toxicity, olanzapine response toxicity, risperidone response	Brain, Cortex, Cerebellum
UPF3B	rs2428212	chrX	N/A	intron	N/A	Mental retardation, x2c syndromic 14, x2c x-linked, non-syndromic x-linked intellectual disability	Cerebellum, Lateral Ventricle

Note: N/A: non-available
