# Supplementary material for: Neurophysiological and Genetic Findings in Patients With Juvenile Myoclonic Epilepsy
Source: Front Integr Neurosci. 2020 Aug 20;14:45. doi: 10.3389/fnint.2020.00045 (PMC7468511; doi:10.3389/fnint.2020.00045)
Supplement: Supplementary file 7 [file Table_7.pdf]

Supplementary Table S7: Enrichment results of the selected top-ranked genes-pathways with Pathway Connector and EnrichR.

**A. Pathway Connector**			
**Number in pathway**	**Gene ID**	**Pathway**	**p-value**
A1	ASPA, FTCD	Histidine metabolism	0.001632
A2	MSH2, AXIN2	Colorectal cancer	0.010527
A3	SLC9A3, SLCO1B3	Bile secretion	0.013637
A4	RPL13, MRPL32	Ribosome	0.04608
**B. EnrichR**			
	SLC9A3, SLC9A7	Sodium/proton exchangers	0.000218
	ASPA, FTCD	Histidine catabolism	0.002382
	APOB, PISD	HNF3A pathway	0.005419
	MSH2, AXIN2	Colorectal cancer proteins	0.01053
	WRN, TNKS	Telomerase regulation	0.01221
	SLC9A3, ECE1	SIDS susceptibility pathways	0.01221
	MCM8, PSMA2	DNA-replication pre-Initiation, S phase, cell cycle checkpoints, mitotic G1-G1/S	0.02045, 0.03198, 0.03464, 0.04488
	SLC9A3, SLC9A7	Transport of inorganic cations/anions and amino acids/oligopeptides	0.02359
	SLC9A3, SLC9A7, SLCO1B3	SLC-Mediated transmembrane transport	0.02483
	MCM8	E2F-enabled inhibition of pre-replication complex formation, unwinding of DNA, CDC6 association with the ORC-origin complex	0.02473, 0.02717, 0.02717

	SLCO1B3	Recycling of bile acids and salts, transport of organic anions	0.02717
	PISD	Phosphatidylethanolamine biosynthesis	0.02960
	ASPA	Alanine and aspartate metabolism	0.02960
	HTR2C	Serotonin receptors-2, Elk-SRF/GATA4 signaling	0.02960, 0.03927
	FTCD	Histidine catabolism	0.01244
	APOB	Chylomicron-mediated lipid transport, platelet sensitization by LDL	0.04168, 0.04168
	TNKS	Telomeres, telomerase, cellular aging and immortality	0.04647
	LATS2	Signaling by Hippo	0.04885
	HTR2C	G-protein signaling through tubby	0.01244
