# Supplementary material for: Neurophysiological and Genetic Findings in Patients With Juvenile Myoclonic Epilepsy
Source: Front Integr Neurosci. 2020 Aug 20;14:45. doi: 10.3389/fnint.2020.00045 (PMC7468511; doi:10.3389/fnint.2020.00045)
Supplement: Supplementary file 8 [file Data_Sheet_1.pdf]

Supplementary Section

1.Details on raw data processing, alignment and variant calling

There is a multi-step process for the WES data analysis. Raw data (Fastq read files) were generated from the sequencing platform via the manufacturer's proprietary software. Fastq files were assessed for quality and trimming using the FastQC version 0.11.7 and FASTX version 0.0.14 toolkits, respectively (Supplementary Table S2). Reads were mapped to their location in the updated human reference genome (hg19/b37) by using the Burrows-Wheeler Aligner (BWA) package, version 0.7.15. The Genome Analysis Tool Kit (GATK) version 3.6-0 was used for realignment of the mapped reads around potential indels (insertion/deletion) sites. GATK ensures the alignment has the minimum number of mismatching bases across the reads and this is to reduce the false-positive SNP calls around indels and determine indels length. Picard (version 2.6.0) was used for annotating duplicate reads that could be the result of PCR artefacts and for not considering them further in the analysis in order to avoid false positive SNPs. Samtools (version 0.1.19) was used for extra Binary Alignment Map (BAM) file operations for fine tuning of the aligned data. GATK's covariance recalibration was used for recalibrating the base quality score (Phred scale) and increasing the accuracy of base quality metrics and variant calls. For each sample, SNP and indel variants were called using the GATK Haplotype Caller and SNP novelty was determined against dbSNP Release147.

2. Details on Risk score prediction using linear logistic regression analysis

This specific package comprises various functions used in the genetic risk prediction studies in order to evaluate the model performance, such as, the plotRoc function. The plotRoc function is used for plotting the ROC curves and for calculating the values of the AUC. Functions of graphical representation of statistical results are also included in the PredictABEL, like, risk distributions (plotRiskDistribution function) and discrimination box plot (plotDiscriminationBox function). The generalized linear model (glm) function of PredictABEL, is substituted with, the bayesglm function via the arm R package (Gelman, 2016). A numeric code (0, 1, 2) was given to the data in order to translate the number of alleles carried; 0 for homozygote reference, 1, for heterozygote and 2, for homozygote alternate.

3. Details on Machine learning data analysis

During each cross-validation round, each sample was taken away and a feature selection was executed on the rest samples of the dataset. Following this, the model was trained and utilized in order to classify the left-out samples. PLINK was used in order to select the features. The leave-one-out cross validation method was used for obtaining the optimal set of variants by testing first the top six variants and then increasing the number of variants for each run until the classification accuracy attained a saturation point without any further improvement. Prediction accuracy, specificity, sensitivity and (MCC) were used so that to evaluate the LOOCV performance (Supplementary Tables S5 and S6). MCC is the balanced measurement for the quality classification that consider the true/false positives and negatives and it gives values within the range of [-1, 1]. Supplementary Figure 1 shows the flow chart of the classification procedure.

4. Details on Enrichment analysis

Pathway Connector is an online tool that provides information based on specific networks, the connections that can appear between various pathways of interest, other complementary

networks that are associated with the pathways of interest and many additional data on how these paths are linked (Minadakis et al., 2019). By providing the list of genes found in our machine learning data analysis, the Pathway Connector (KEGG 2016) automatically applied enrichment analysis (Supplementary Table S7). We obtained the top-scored pathway lists for the specific network indicating the p-value of each group of genes that found to be involved in a common pathway so that we can use the ones that had a significant score ($p<0.05$). In addition, it provides schematically genes that were found to share a common network and the connections of the pathways found (Minadakis et al., 2019). In a similar way, the EnrichR tool (KEGG 2019) (Chen et al., 2013;Kuleshov et al., 2016) revealed a set of common shared pathways along with the p-values with the implicated genes being reported (Supplementary Table S7).

5. Supplementary Figure Legends

Supplementary figure 1: Classification process flow chart

Step 1- Defines the two groups of participants; 16 polyphasic and 14 non polyphasic subjects. Step 2 - Selection of x set of variants to be used during the LOOCV procedure. Step 3 - Initiation of the LOOCV by removing one sample from the dataset. Step 4 - Odds ratio analysis on the remaining samples by using PLINK. An x number of the top significant variants are selected to be used for next step. Step 5 – Training of the linear regression model with the data and features used for the repetition. Step 6 - Repetition of the LOOCV process (Steps 3-5) for each of the 30 samples and calculation of statistics. Step 7 – Repetition of Steps 2-5 for every value of x

Supplementary figure 2: Classification process results.

The graph illustrates the statistics for each LOOCV run across the different values of the top significant variants that were selected for the validation. Blue bars show the LOOCV prediction accuracy; orange bars, the sensitivity; grey bars, the specificity; and the yellow line demonstrates the Matthew's correlation. Optimal results (>80% prediction accuracy, sensitivity and specificity) are observed for selection of 16 variants for every LOOCV run, as shown by the peak in the Matthew's correlation curve.

Supplementary figure 3: Receiver operating characteristic plot corresponding to the current classification scheme.

The ROC curves resulted in area under the curve (AUC) with a value of 0.871.

Supplementary figure 4: Total SNVs found in the two-case scenario.

The pie chart illustrates the number of variants and the different types of impact of all variants found in the investigation from the examined samples.

Supplementary figure 5: Pathways implicated with the various genes found in the two-case scenarios with the Pathway Connector.

(A)Pathway-to-Gene Network for the top scored selected pathways that derive from the Enrichment Results. (B) Pathway-to-pathway network of the top scored selected pathways that derive from the enrichment results. The edge weight characterizes the number of common genes between the pathways. Both images were obtained from the Pathway Connector.

6. Supplementary Table Legends

Supplementary Table 1: First-degree relatives and controls age range.

Supplementary Table S2: Details on the sequencing depth and coverage for each individual of each trio.

Supplementary Table S3: Sanger sequencing validation for the candidate gene *SYT14.*

Supplementary Table S4: Common genes reported in Epilepsy, ALS and MD gene panels.

Supplementary Table S5: Candidate variants found for polyphasic vs non-polyphasic molecular classification selected from LOOCV.

Supplementary Table S6: Candidate variants of polyphasic vs non-polyphasic molecular classification.

Supplementary Table S7: Enrichment results of the selected top-ranked genes-pathways with Pathway Connector and EnrichR.

References

Chen, E.Y., Tan, C.M., Kou, Y., Duan, Q., Wang, Z., Meirelles, G.V., Clark, N.R., and Ma'ayan, A. (2013). Enrichr: interactive and collaborative HTML5 gene list enrichment analysis tool. *BMC Bioinformatics* 14**,** 128.

Gelman, A.a.Y.S. (2016). arm: Data Analysis Using Regression and Multilevel/Hierarchical Models. R package version 1.9-3. .

Kuleshov, M.V., Jones, M.R., Rouillard, A.D., Fernandez, N.F., Duan, Q., Wang, Z., Koplev, S., Jenkins, S.L., Jagodnik, K.M., Lachmann, A., Mcdermott, M.G., Monteiro, C.D., Gundersen, G.W., and Ma'ayan, A. (2016). Enrichr: a comprehensive gene set enrichment analysis web server 2016 update. *Nucleic Acids Res* 44**,** W90-97.

Minadakis, G., Zachariou, M., Oulas, A., and Spyrou, G.M. (2019). PathwayConnector: finding complementary pathways to enhance functional analysis. *Bioinformatics* 35**,** 889-891.
