# Supplementary figures and images for: Neurophysiological and Genetic Findings in Patients With Juvenile Myoclonic Epilepsy
Source: Front Integr Neurosci. 2020 Aug 20;14:45. doi: 10.3389/fnint.2020.00045 (PMC7468511; doi:10.3389/fnint.2020.00045)

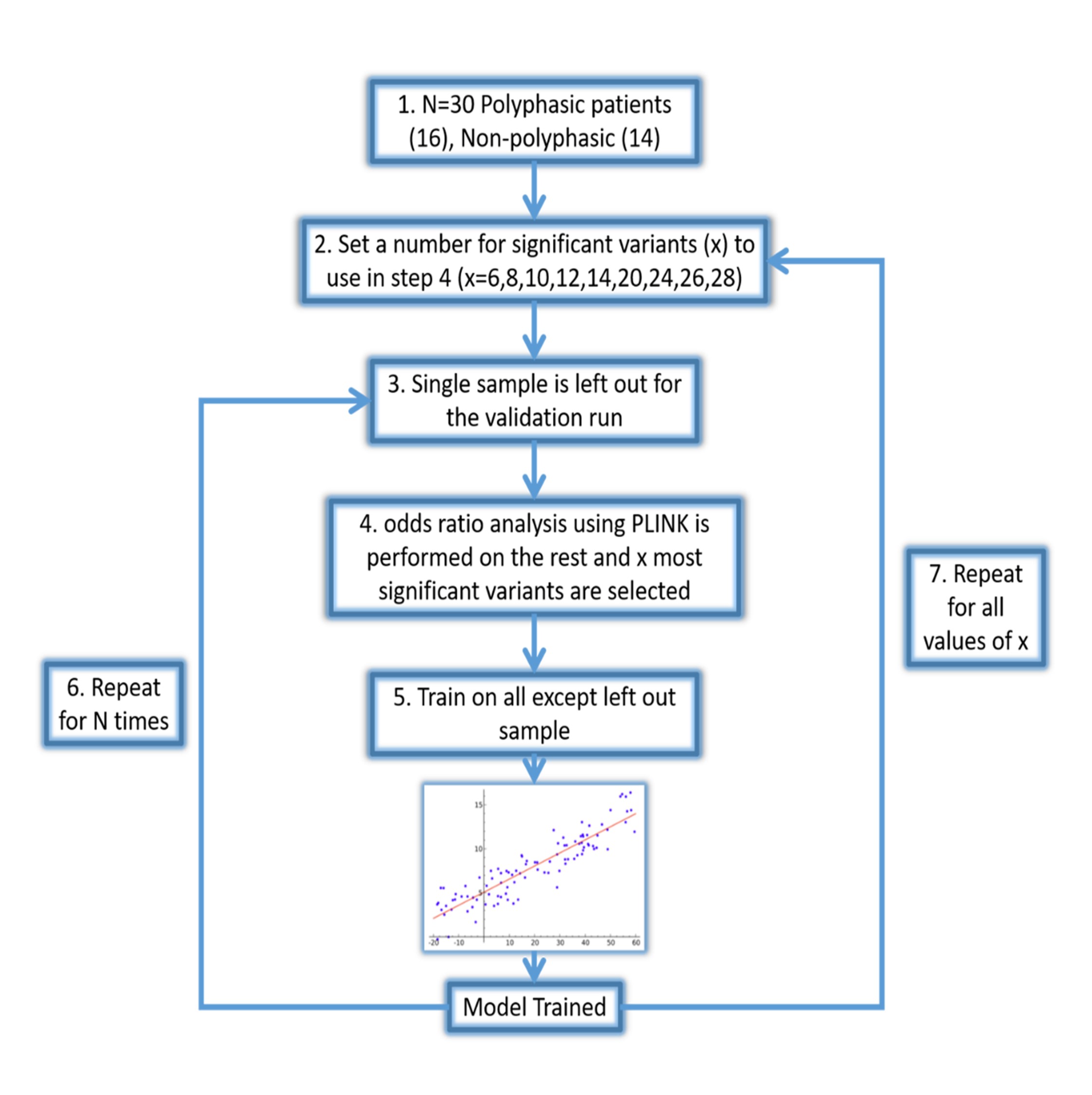

Supplement: Supplementary file 9 [file Image_1.JPEG]

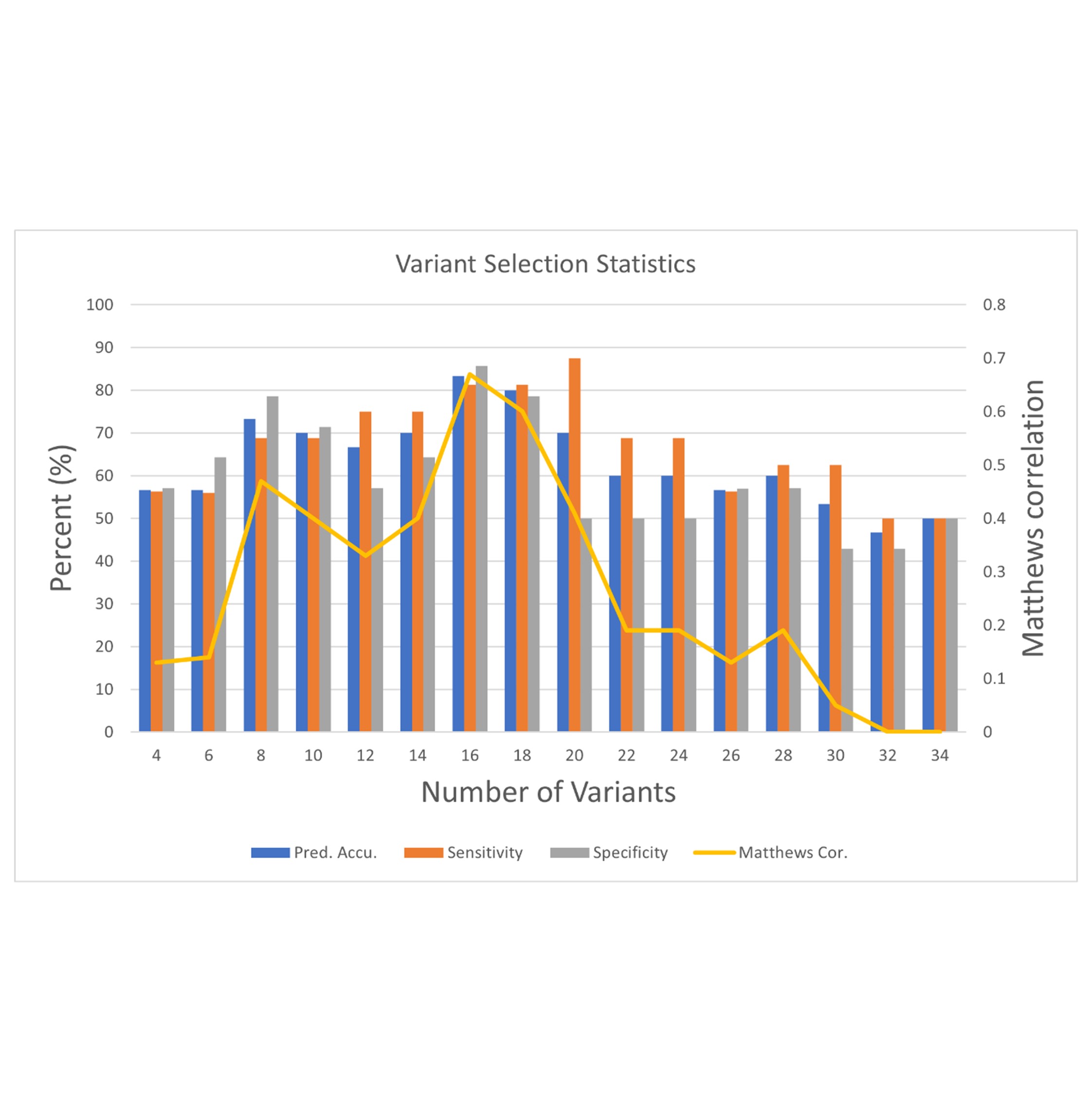

Supplement: Supplementary file 10 [file Image_2.JPEG]

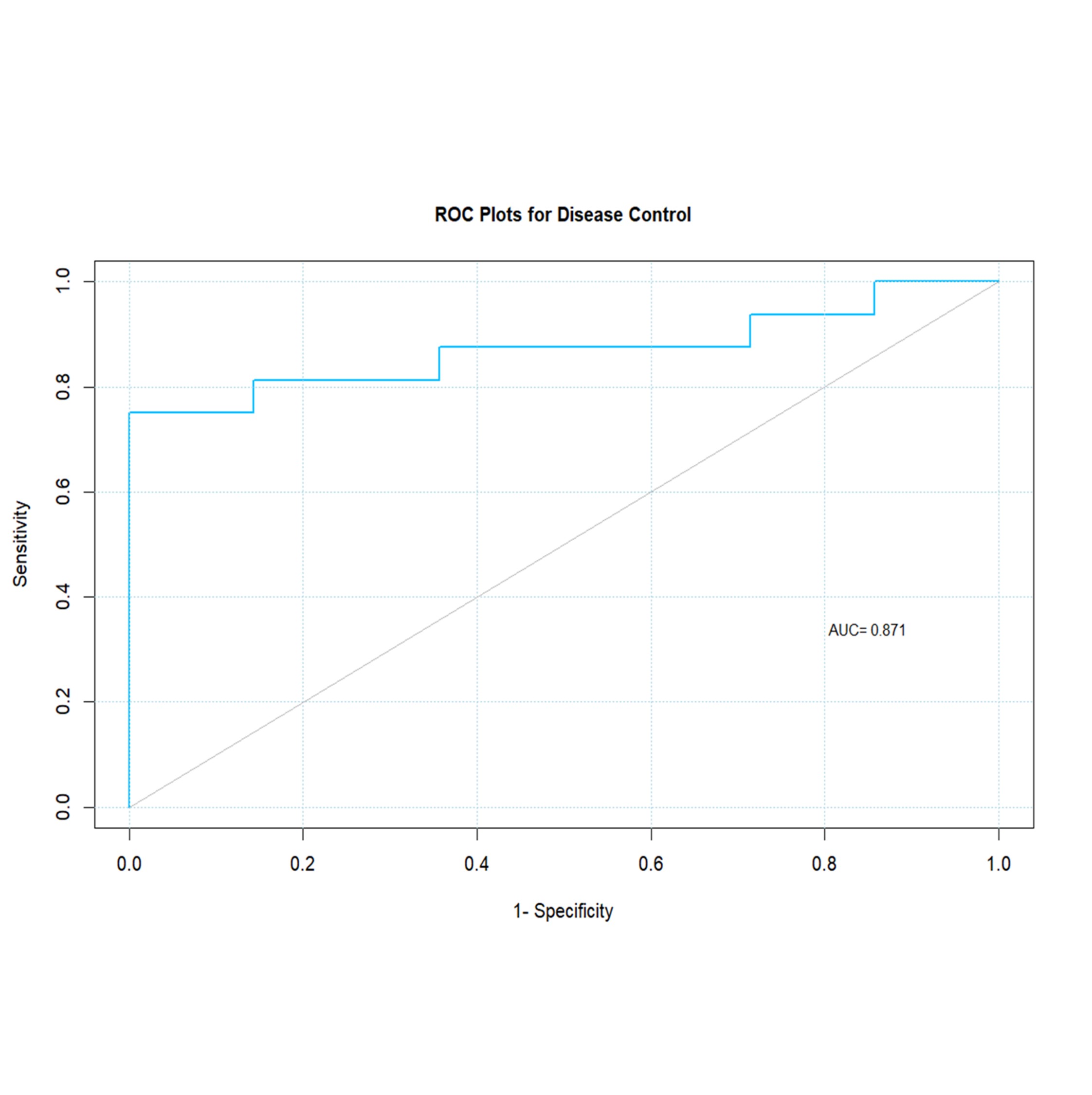

Supplement: Supplementary file 11 [file Image_3.JPEG]

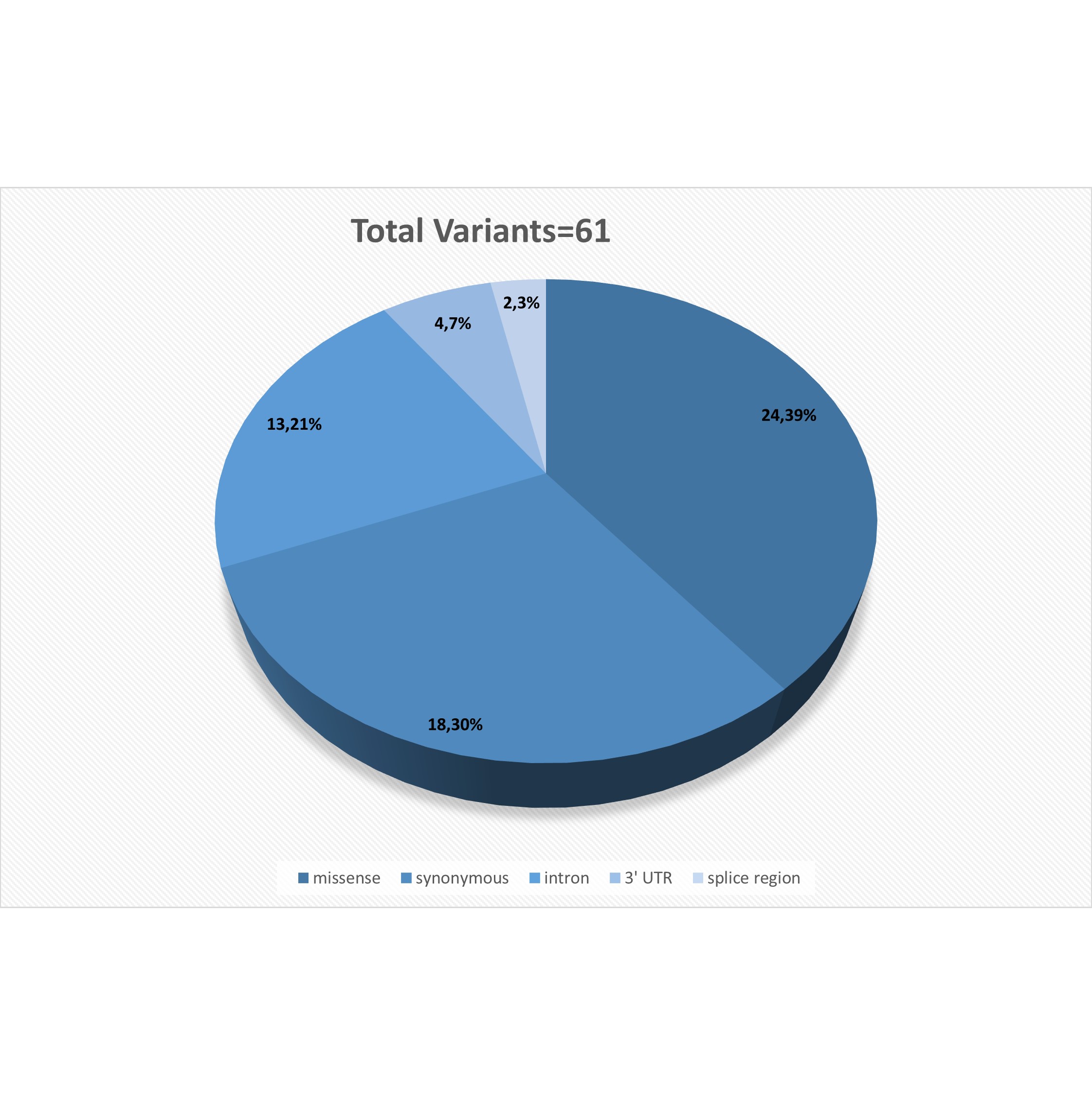

Supplement: Supplementary file 12 [file Image_4.JPEG]

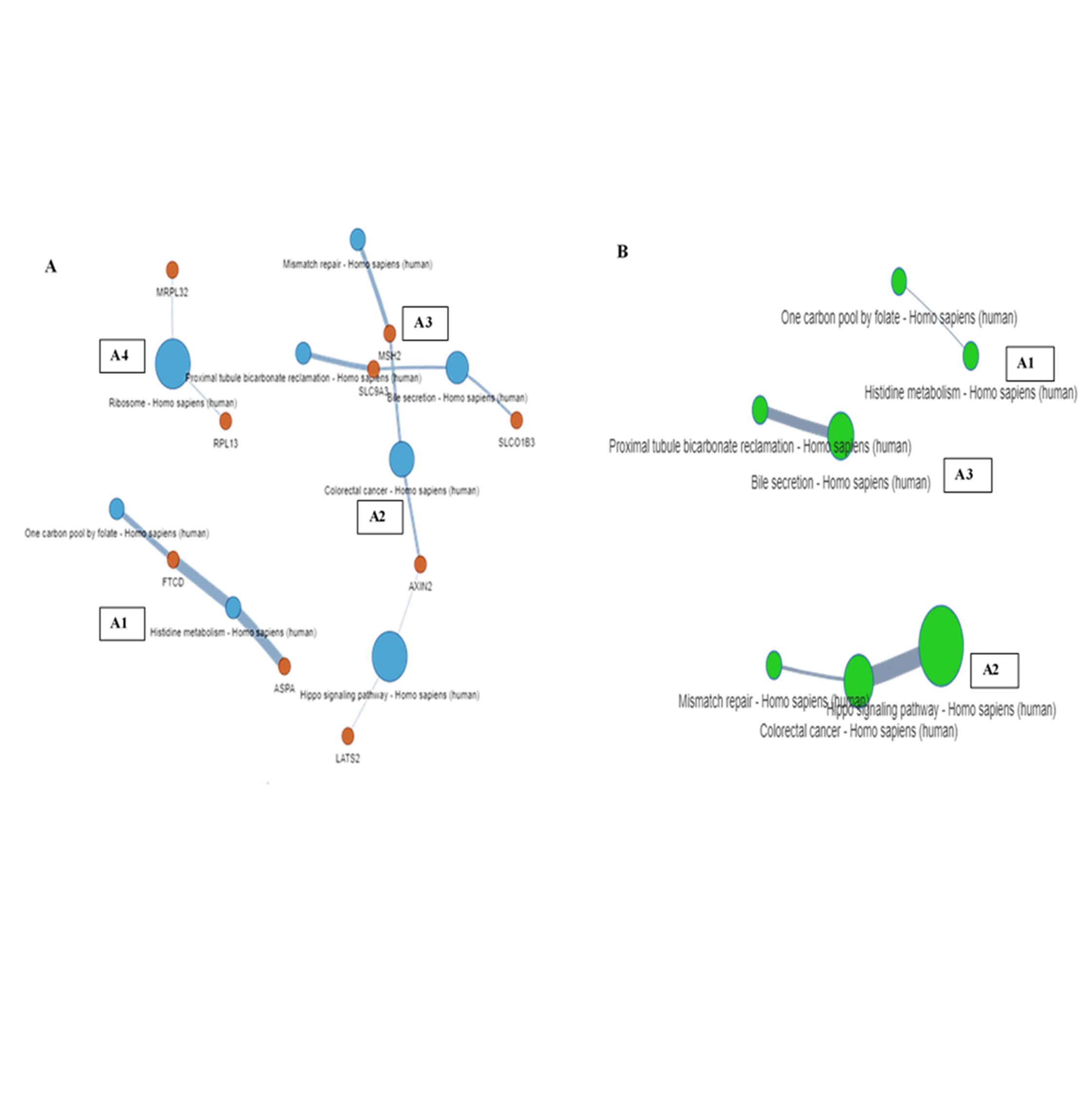

Supplement: Supplementary file 13 [file Image_5.JPEG]
